# The CXCL12/CXCR4 axis in breast cancer-associated fibroblasts: a transcriptome analysis and systematic literature review

**DOI:** 10.3389/fbioe.2026.1920541

**Published:** 2026-10-12

**Authors:** Sergey Vladimirov, David Sergeevichev, Maria Bogachek, Alina Kazakova, Anastasia Leonteva, Vladimir Richter, Anna Nushtaeva

**Affiliations:** 1 Scientific Center of Genetics and Life Sciences, Sirius University of Science and Technology, Sirius, Russia; 2 Meshalkin National Medical Research Center, Novosibirsk, Russia; 3 Laboratory of Biotechnology, Institute of Chemical Biology and Fundamental Medicine, Siberian Branch of the Russian Academy of Sciences, Novosibirsk, Russia

**Keywords:** autocrine and paracrine regulation, breast cancer, cancer-associated fibroblast, CXCL12/CXCR4, SDF-1α, SPP1(OPN), TGF-β1, tumor microenvironment

## Abstract

**Background:**

Cancer-associated fibroblasts (CAFs) represent a pivotal component of the breast cancer microenvironment. In a paracrine model, CAFs secreted the chemokine CXCL12 (SDF-1α), which bound to CXCR4 and activated the PI3K/AKT and MAPK signaling pathways, stimulating their migration and invasion. TGF-β1 induces myofibroblast differentiation of CAFs by acting through the TGFBR2/ALK5 receptor complex and phosphorylating SMAD2/3, thereby increasing the expression of ACTA2 and FAP. An autocrine loop has been demonstrated in CAF, whereby TGF-β1, via SMAD3, induces the transcription of CXCR4, and subsequently, CXCR4, by binding to the same cell, maintains the myofibroblast phenotype. However, a systematic comparison of paracrine and autocrine variants has not yet been conducted in the context of CAF subtypes.

**Methods:**

A secondary analysis of 101 bulk RNA-seq samples was performed, with classification into healthNF, riskNF, cancerNF, and CAF S1-S4 using correlation analysis (Spearman), differential expression (DESeq, FDR <0.05), GSEA (FDR <0.25), and a systematic review of the literature (2005–2026).

**Results:**

The PCA and the contingency table analysis enabled the description of two patterns of Cancer-associated fibroblasts subtype distribution across clinical and morphological groups: healthNF → cancerNF → CAF and healthNF → riskNF → CAF. Elevated levels of CXCR4 were identified exclusively in CAF (22.7%, FDR = 5.98 × 10^−4^) and were associated with the CAF-S3 and CAF-S4 fibroblast phenotypes. At CXCR4^High^ levels, the correlation between CXCL12 and TGFB1 became negative, while the correlation between CXCL12 and ITGAV, FAP, and ITGB1 remained positive. This finding suggests a possible shift from TGF-β-dependent to CXCR4-mediated regulation of CXCL12. GSEA revealed an association of CXCR4^High^ -CAF with enrichment of gene sets related to the interferon response, inflammation, and apoptosis, as well as with decreased enrichment of proliferation sets, EMT, and TGF-β signaling. A recent systematic review revealed that only 8.6% of publications contain experimental data supporting an autocrine loop, 17.1% describe a paracrine model, and 74.3% do not distinguish between the signaling types.

**Conclusion:**

It is hypothesised that the CXCR4^High^-CAF transcriptome profile is compatible with a biologically plausible theory of transition between paracrine and autocrine regulatory modes and inflammatory cell states. *In vitro* validation is a prerequisite for establishing a causal relationship.

## Graphical Abstract

**Figure:**
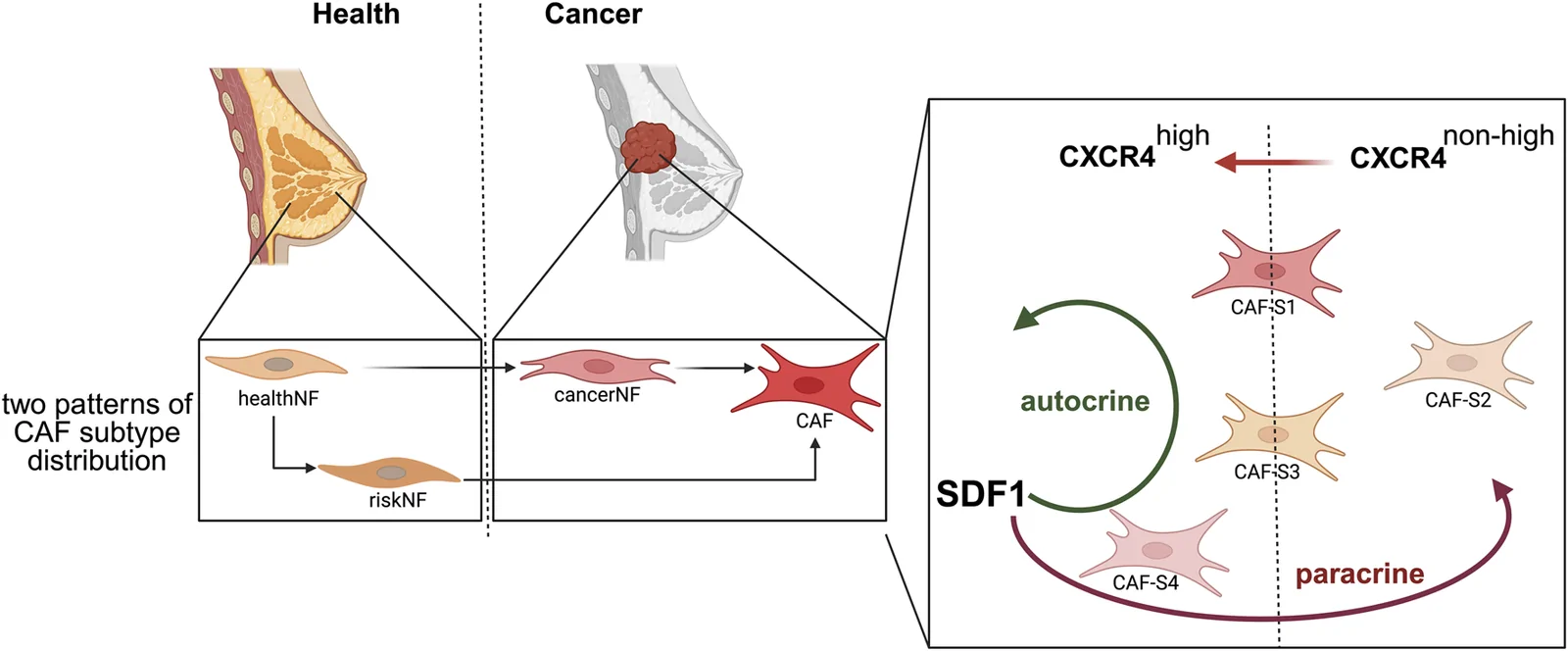
The two trajectories of fibroblast activation.

## 1 Introduction

Fibroblasts are a type of cell that is characterized by its non-epithelial and non-immune properties. These cells are typically of mesenchymal origin (Kalluri, 2016). In the context of diseases, fibroblasts have been shown to play a pivotal role in tissue fibrosis and wound healing (Jiang et al., 2023). Fibrotic diseases and solid cancers are distinguished by persistent inflammation and tissue damage. It is a widely accepted fact that fibrosis is regarded as a hallmark of cancer (Salvatore et al., 2025; Ricco et al., 2026).

There is a considerable body of evidence indicating that fibroblasts play a pivotal role in the promotion of fibrosis and extracellular matrix (ECM) compaction, thereby accelerating disease progression through a multitude of mechanisms (Shiraki et al., 2022; Boeker et al., 2025; Lu et al., 2025). In the complex microenvironment (TME) of the mammary gland, stromal cells create a favorable “soil” for the occurrence of breast cancer (BC) (Alexander et al., 2020; Nushtaeva et al., 2023). Stromal-epithelial interactions have been demonstrated to play an instrumental role in the formation of normal milk ducts and in the complete differentiation of lobular alveoli (Kim et al., 2005). This dynamic process is of significant importance in the context of carcinogenesis. An aberrant reaction of the stroma and epithelium results in the failure of epithelial cells to polarize, thereby leading to uncontrolled cell proliferation and, consequently, carcinogenesis.

Cancer-associated fibroblasts (CAFs) are a type of fibroblast commonly found within and around tumors. This group comprises native, normal fibroblasts and activated, proliferating fibroblasts, as well as fibroblasts that have been recruited in response to a stimuli induced by cancer cells (Lan et al., 2025; Ermakov et al., 2022). To date, CAFs have been detected and studied in almost all human organs where carcinogenesis is possible. However, the scope of studies and their results vary greatly depending on the specific type of cancer (Boeker et al., 2025; Wang H. et al., 2026). In certain neoplasms, such as those of the breast and pancreas, the CAFs have been the subject of extensive study and characterization (Yang D. et al., 2023; Qu et al., 2025; Cords et al., 2024).

There remains a paucity of consensus on nomenclature for cancers in general, and consequently for individual fibroblast subtypes, which hinders identification of their respective functions (Ilyina et al., 2025; Fan et al., 2025; Cords et al., 2023). The most widely used nomenclature classifies CAFs into three main subgroups: myofibroblast-like CAFs (myCAFs), immunomodulatory CAFs (iCAFs), and antigen-presenting CAFs (apCAFs) (Lan et al., 2025; Yang D. et al., 2023; Caligiuri et al., 2023). A notable limitation of these classifications is their inability to clearly distinguish between fibroblast populations that promote or impair tumor growth (Boeker et al., 2025). Recent studies employing single-cell and spatial multi-omics methodologies have yielded efficacious strategies for the characterization of spatial organization and the classification of the TME (Pelon et al., 2020; Liu et al., 2025). Costa et al. (2018) proposed a classification of BC CAFs into four subtypes based on a combination of six markers (ACTA2/α-SMA, FAP, ITGB1/CD29, PDGFRβ, CAV1, S100A4/FSP1) (Bogachek et al., 2025). Quiescent fibroblasts present in non-tumor tissues exhibited characteristics analogous to those of CAF-S2 (ACTA2^low^/ITGB1^low^/PDGFRβ^low^) and CAF-S3 (ACTA2^low^/ITGB1^med^/PDGFRβ^med^). The functions of these cells were analogous to those of normal resident fibroblasts. Conversely, myofibroblasts detected exclusively in tumors and metastatic lymph nodes exhibited the phenotypes of CAF-S1 (ACTA2^high^/FAP^high^) and CAF-S4 (ACTA2^high^/FAP^low^/ITGB1^high^). CAF-S1 exhibited characteristics associated with both ECM adhesion and wound healing, in addition to immunosuppressive effects. In contrast, CAF-S4 exhibited features of perivascular contraction, which promoted metastasis (Nurmik et al., 2020; Pawar et al., 2025). The extant research has confirmed the presence of both extracellular matrix-rich CAF-S1 subsets and reduction-rich CAF-S4 subsets in a range of oncological conditions, including pancreatic, colorectal, breast, lung and head and neck cancer. These findings have been observed in both human and mouse tumor models (Bartoschek et al., 2018; Lambrechts et al., 2018; Hornburg et al., 2021; Peltier et al., 2022; Pawar et al., 2025). Despite the utilization of disparate methodological approaches, contemporary multiomic studies have elucidated significant overlap in the classifications obtained. Single-cell sequencing of BC has revealed that the CAF-S1 population is heterogeneous, comprising subclusters that correspond to two distinct populations of cells: myCAF-like cells and iCAF-like cells. myCAF-like cells are characterized by high expression of α-SMA and matrix genes, while iCAF-like cells exhibit inflammatory cytokine expression (Mhaidly and Mechta-Grigoriou, 2020; Chhabra and Weeraratna, 2023). Conversely, the CAF-S4 subtype is characterized by an accumulation of genes linked to myofibroblast differentiation, suggesting its classification as a variant of myCAF with augmented invasive potential. Consequently, the S1–S4 classification functions as a marker-based classification that also encompasses less well-characterized groups of normal-like fibroblasts, whilst myCAF/iCAF/apCAF provides a functional interpretation within these populations. Whilst the CAF-S1-S4 classification provides a more detailed breakdown, it does so at the expense of reduced functional specificity.

CAFs secrete a variety of factors, including but not limited to: CXCR4, TGF-β1, PDGF, HGF, and components of the ECM. These factors act in a paracrine manner on tumor, endothelial and immune cells. The presence of CAFs has been demonstrated to be associated with a number of biological processes, including the stimulation of tumor cell proliferation and migration, and the promotion of angiogenesis. In addition, CAFs have been shown to exert a suppressive effect on cytotoxic T cells (Orimo et al., 2005; Kalluri, 2016; Leonteva et al., 2025). Transforming growth factor-beta (TGF-β1), a multifaceted cytokine, is secreted by CAFs in a latent form and is subsequently activated by proteases or integrins within the extracellular space. The process of active TGF-β1, via the TGFBR2/ALK5 complex, triggers the phosphorylation of SMAD2/3. This, in turn, leads to their binding to SMAD4, and their subsequent translocation into the nucleus. The result of these processes is the induction of the transcription of the ACTA2, FAP, and CXCL12 genes (Kojima et al., 2010; Massagué, 2012; Caja et al., 2018; Liu et al., 2008). In the early stages of carcinogenesis, the growth of epithelial cells is suppressed by TGF-β, which activates cyclin-dependent kinases (CDKs) and results in the induction of CDK inhibitors, including p15 and p21 (Cipriano et al., 1998; Datto et al., 1995). As oncogenic mutations accrue, this branch of signaling is subsequently lost. However, pro-invasive effects, including epithelial–mesenchymal transition and immunosuppression, endure (Massagué, 2012; Caja et al., 2018). Chemokine receptor CXCR4, categorized as a class A Gi-coupled receptor, is activated by the chemokine ligand, termed “CXCL12” and also referred to as “SDF-1α”. Activation of CXCR4 has been observed to lead to the dissociation of Gαi (which inhibits adenylate cyclase) and Gβγ (which activates PI3K/AKT and PLCβ/PKC), as well as the β-arrestin-dependent ERK1/2 pathway. In a paracrine model, the binding of CXCL12 to CXCR4 expressed on tumour cells has been demonstrated to stimulate their directed (Orimo et al., 2005; David et al., 2018; Zhou et al., 2018). In addition to the paracrine function, experimental data indicate the existence of an autocrine loop involving the chemokine, CXCR4, and its receptor, CXCR4, directly in CAF. Kojima et al. (2010) demonstrated that in primary cultures of mammary fibroblasts, TGF-β1 induces CXCL12 transcription via SMAD3. Secreted CXCL12 binds to CXCR4 on the same cell, thereby maintaining the myofibroblast phenotype (increased ACTA2, FAP), and forming a positive feedback loop. Rovito et al. (2016) discovered elevated levels of co-expression of CXCR4 and SDF-1α in breast cancer samples that had metastasized to the lymph nodes. They cited the findings of Kojima et al. (2010) as substantiating evidence for an autocrine loop, though they did not conduct independent experimental verification in CAF.

Thus, direct experimental evidence for an autocrine loop in CAFs is presented only in a single study by Kojima et al. (2010), and no systematic comparison of paracrine and autocrine variants in the context of CAF differentiation has been performed. A comprehensive evaluation of the existing secondary transcriptomic data analysis and systematic literature review is imperative to ascertain whether elevated CXCR4 expression is associated with altered gene co-expression patterns that are consistent with a transition from paracrine to autocrine regulation of SDF-1α in mammary CAFs classified into the S1-S4 subtypes.

## 2 Methods

A set of criteria was utilized to evaluate the quality of the studies incorporated within the systematic review, with consideration given to the heterogeneity of study designs. The following criteria were taken into account: the clarity of the identification of the CAFs population; the presence of validated CAF-specific markers or signatures; the reproducibility of the detection method; the presence of adequate controls; and a clear indication of the type of signaling interaction (autocrine, paracrine) or its absence. The decision to adopt this approach was motivated by the substantial discrepancies observed among functional, transcriptomic and associative studies, which rendered the utilization of a singular formal tool for evaluating the risk of bias across all studies impracticable.

Concomitantly, a number of potential biases must be considered, including publication bias in favor of positive results, heterogeneity in experimental and clinical designs, differences in the endpoints utilized, and incomplete experimental validation in certain studies. The combination of these factors precludes the possibility of a quantitative comparison of results, thus rendering a qualitative thematic synthesis of the data the preferred approach.

### 2.1 Study design

This study presents an integrative transcriptome analysis of publicly available bulk RNA-seq data from mammary stromal fibroblasts (NF) and CAF subtypes of breast cancer, accompanied by a systematic literature review. The work was conducted in three sequential stages. A systematic review of the chemokine receptor CXCR4 and its associated axis in CAFs was performed in accordance with the Preferred Reporting Items for Systematic Reviews and Meta-Analyses (PRISMA) 2020 guidelines (Page et al., 2021) (Supplementary Figure S1). The second step involves the search and curation of human breast fibroblast transcriptome data from the NCBI Sequence Read Archive (SRA) and Gene Expression Omnibus (GEO). The third phase involves the quantification of expressions, sample classification, and statistical analysis.

### 2.2 Systematic literature review strategy

The systematic literature review strategy comprised the formulation of the research question, the specification of literature search sources and time period, the definition of publication inclusion and exclusion criteria, the study selection process, and qualitative data synthesis. A detailed description of the methodology is provided in the Supplementary Materials.

### 2.3 Transcriptomic data collection strategy

#### 2.3.1 Search sources and period

To generate a representative sample of transcriptomic data and ensure the traceability of biological samples, a software algorithm for automated metadata collection from NCBI repositories was developed. This algorithm was implemented using the BioPython library and the Entrez Programming Utilities (E-utilities) API.

#### 2.3.2 Search query structure

A search query was utilized to identify relevant datasets. The search query targeted fibroblast cell populations capable of acquiring a CAF-like phenotype in the breast tumor microenvironment. The comprehensive search query was as follows: *(“fibroblast”[All Fields] OR “fibroblasts”[All Fields] OR “CAF”[All Fields] OR “CAFs”[All Fields]) AND (“breast”[All Fields] OR “mammary”[All Fields]) AND (“Homo sapiens”[Organism] AND “transcriptomic”[Source] AND “biomol rna”[Properties] AND “filetype fastq”[Properties]).*

This identified 1,015 SRA records, from which the corresponding BioSample, GEO Sample (GSM), and GEO Series (GSE) identifiers were subsequently extracted.

#### 2.3.3 Metadata extraction and aggregation

For each SRA ID that was obtained, an extensive XML summary was extracted using a batch processing method implemented in Entrez.esummary. Unique biological sample identifiers (BioSample Accession, SAMN/SAMEA formats) were algorithmically extracted from the ExpXml field of each record, thereby enabling the linkage of specific sequencing datasets to their biological source. Subsequently, cross-queries were executed to establish a link between the raw sequencing data and the results of their analysis within the context of published series. SAMN/SAMEA identifiers were then compared with the BioSample database to extract metadata on experimental conditions, tissue identity, and cell types. Sample description IDs from the GEO database (GSM) were extracted from the BioSample ID fields. Based on the obtained GSM identifiers, corresponding batch identifiers (GSEs) were identified by searching the GDS (GEO DataSets) database. For each batch, complete metadata was obtained, including project names, sequencing platform annotations, and relationships between samples within a batch. The final data structure was formed by combining information at four levels: project/batch (GSE) → sample (GSM) → biological sample (BioSample) → sequencing data (SRA).

#### 2.3.4 Semantic filtering and exclusion criteria

During the aggregation stage, a set of customizable exclusion filters were applied based on the following attribute values: source_name, tissue, cell_line, cell_type, genotype, and treatment. Given that metadata in NCBI repositories is manually populated by authors, a manual audit of these fields was conducted. Samples containing the following were excluded from the study:

- off-target biological material (MCF-7, MDA-MB-231, T47D tumor lines; epithelial cells; endothelial cells; organoids);
- fibroblasts from non-target locations (skin, bone marrow, brain metastases).
- samples with severe chemical or genetic modifications (treatment with MCL-1 antagonists, transfection with specific siRNA) that critically alter the transcriptome profile
- data obtained by single-cell RNA-seq to avoid bias due to the specificity of single-cell measurements.

Accordingly, an iterative exclusionary lexicon was formulated subsequent to this analysis. The result was a comprehensive JSON metadata file containing complete hierarchical information about each sample. All data loading steps were accompanied by operational status logging (downloaded, processed, errors) to ensure reproducibility. Consequently, SRA identifiers for 264 samples were selected, and these samples were subsequently submitted to the manual curation stage.

#### 2.3.5 Sample inclusion and exclusion criteria

The selection of samples for analysis was based on a manual data curation stage. For each sample (SRR ID), a detailed study of the study design was conducted based on original publications and repository metadata (GSE, PMCID). This approach facilitated the development of an expert dictionary, wherein each identifier is assigned a biological type based on the sample preparation conditions.

The inclusion criteria encompassed the clinical and morphological characteristics of the samples, which facilitated their classification into the following cohorts:

- healthNF (fibroblasts from breast tissue of healthy women);
- riskNF (fibroblasts from breast tissue of women with a detected BRCA1 gene mutation after prophylactic reduction mammoplasty);
- cancerNF (fibroblasts from tumor-free tissue of breast cancer patients);
- CAF (cancer-associated fibroblasts derived from breast cancer samples).

Exclusion criteria:

- immortalized cell lines;
- fibroblasts obtained from dermal biopsies;
- samples with an uncertain experimental design.

Of the 264 samples, 173 were included in further analysis after passing expert review according to the inclusion criteria and having a full match between SRA identifiers and GEO repository metadata.

#### 2.3.6 Sample selection by type of sequencing

Of the 173 samples, 101 were sequenced using the paired-end protocol, while 72 were processed using the single-end protocol. In order to guarantee sample homogeneity and to minimize technical variability, only paired-end samples (n = 101) were included in the analysis. The rationale underlying this approach is as follows:

- Single-end and paired-end libraries are prepared using different protocols, which can create a significant batch effect, leading to the clustering of samples based on preparation method rather than biological properties.
- Paired-end reads provide more accurate mapping, especially in regions with duplications and alternative splicing.
- The paired-end group constitutes a majority (101 vs. 72), which improves the reproducibility of the results.

### 2.4 Bioinformatics data processing pipeline

The raw reads were loaded using SRA Toolkit v2.11.3 (fastq-dump--split-files--gzip). Expression quantification was performed using Kallisto v0.46.2 (Bray et al., 2016), employing a pre-built human transcriptome index constructed from the GRCh38.p14 reference RNA sequences (GCF_000001405.40_GRCh38.p14_rna.fna). The kallisto_quant procedure was executed for each sample with the default preset for paired-end reads. The results were stored in a tabular format, with columns containing transcript identifiers, length, effective length, expression estimates in TPM units, and estimated read counts (est_counts). The final expression matrices (abundance.tsv) for each sample were renamed according to the sample’s SRA identifier and moved to a single results directory.

Repeat quality control of raw reads (FastQC, MultiQC) was not performed. The analysis is predicated on data that passed quality control (QC) in the original studies. This limitation must be taken into account when interpreting the results.

### 2.5 Data preprocessing and normalization

The quantitative results (TPM) were filtered by gene prefixes that did not carry information: LOC, RN7S, RPPH, RNVU, RNU, SNOR, SCAR, BCYRN, LINC, and MIR.

Metadata analysis of the biological samples indicated that differences in sequencing protocols represented a major potential source of technical variability. To reduce this source of batch-effect, all samples generated using single-end sequencing were excluded from the analysis. All subsequent analyses were performed exclusively on the homogeneous set of paired-end samples, which improved quantification accuracy and enhanced comparability across sequencing libraries.

To place data originating from different sources on a common analytical scale, expression values were transformed using log2 (TPM+1). This transformation reduced the influence of heteroscedasticity, a characteristic feature of RNA-seq expression data.

Marker-expression levels were categorized using a parametric Mean ±1 SD approach applied to the normalized data. This approach adaptively accounts for gene-specific variability across the pooled cohort, thereby reducing the influence of residual between-study fluctuations and limiting false-positive classification of samples into expression categories, such as High or Neg.

### 2.6 Transcriptome data analysis methodology

The transcriptomic data analysis comprised several sequential steps: categorization of expression levels, including fibroblast subtyping into the S1-S4 subtypes; differential gene expression analysis; principal component analysis (PCA); contingency table construction with standardized residual analysis; correlation analysis; and gene set enrichment analysis (GSEA). A detailed description of the methodology is provided in the Supplementary Materials.

### 2.7 Protein-protein interaction analysis (STRING)

Protein-protein interaction analysis was performed using the STRING v12 database (Szklarczyk et al., 2025). The input data encompassed CAF marker genes (ACTA2, FAP, ITGB1, PDGFRB, CAV1, S100A4) and genes from the CXCL12/CXCR4 axis (CXCL12, CXCR4, TGFB1, ITGAV, ITGB3, CD44). The following parameters were utilized: organism = *Homo sapiens*; confidence threshold = medium (0.400). Gene Ontology (Biological Process) annotation was performed using the STRING platform.

### 2.8 Statistical analysis

All statistical calculations were executed using Python 3.10. For differential expression analysis, the DESeq2 package was utilized via the rpy2 interface. Multiple testing correction was performed using the Benjamini-Hochberg method (FDR). The significance level for all tests was set at p < 0.05, unless otherwise specified (GSEA: FDR <0.25).

## 3 Results

### 3.1 The significance of the CXCR4/CXCL12 axis in SDF-1α secretion

After the removal of duplicates and the examination of eligibility, a total of 34 publications covering the period from 2005 to 2026 were included in the systematic review. Of these, only 3 studies (8.6%) clearly indicated or experimentally substantiated the presence of an SDF-1α/CXCR4 autocrine signaling loop in CAFs. Eck et al. (2009) proposed a potential autocrine loop, as indicated by the elevated expression of SDF-1α and CXCR4 in CAFs. Kojima et al. (2010) provided direct evidence for an autocrine loop in which TGF-β and SDF-1α mutually induce each other in CAFs. Rovito et al. (2016) corroborated the existence of an autocrine signaling circuit in CAFs, citing the work of Kojima et al. (2010).

Six publications (17.1%) provide a comprehensive account of the paracrine role of the chemokine receptor axis. In these publications, SDF-1α, which is secreted by CAFs, acts on CXCR4 of tumor cells or the endothelium (Orimo et al., 2005; Suo et al., 2015; Kalpana et al., 2019; Scordamaglia et al., 2022; Jang et al., 2023; Wang L. et al., 2026a).

In the remaining 26 publications (74.3%), the signaling type is not specified, and the role of CXCR4 is considered primarily in the context of tumor cell migration, angiogenesis, immunosuppression, or therapeutic targets, without differentiating between paracrine and autocrine mechanisms.

The majority of the papers focus on the CXCL12/CXCR4 axis in CAFs, accounting for over 50% of the included studies, which were published from 2023 to 2026. This signifies a notable increase in research interest concerning this signaling axis. However, none of the publications subsequent to 2016 have conducted direct testing of the autocrine loop in fibroblasts. Conversely, research endeavors are currently directed towards the blockade of CXCR4 as a means to impede paracrine communication between CAFs and tumor cells.

A qualitative meta-analysis methodology (thematic synthesis) was employed to assess the strength of the evidence supporting an autocrine role for SDF-1α/CXCR4 in CAFs. Direct evidence in the form of experimental confirmation of a loop is provided by only two studies (Kojima et al., 2010; Rovito et al., 2016). These findings suggest that SDF-1α, through its binding to CXCR4 on CAFs, induces the expression of TGF-β. Consequently, this results in an augmentation of SDF-1α production, thereby completing the autocrine loop. The study by Eck et al. (2009) offers an exploration of indirect evidence, including the increased co-expression of ligand and receptor in CAFs and the possibility of an autocrine loop as a hypothesis. In the vast majority of subsequent studies, including those with a high level of evidence (single-cell RNA-seq, spatial transcriptomics), the authors do not analyze or discuss autocrine signaling in CAF. Furthermore, the studies are significantly heterogeneous in design (*in vitro*, *in vivo*, transcriptome analysis, clinical samples), detection methods (PCR, IHC, flow cytometry), and endpoints. The risk of publication bias, defined as the tendency of reporting positive results more frequently, is evaluated as moderate. However, the bias in favor of the paracrine model is evident, as most studies do not even attempt to investigate the autocrine component.

### 3.2 Transcriptome profiling and classification of samples into functional subtypes

#### 3.2.1 Distribution of samples by subtypes

The classification of 101 samples into functional subtypes using an algorithm adapted from Costa et al. (2018) revealed the following distribution: subtype S2 - 47 samples (46.5%), S1 - 33 (32.7%), S3 - 13 (12.9%), S4 - 8 (7.9%). In the PCA plot, samples with the S2 phenotype formed a central cluster, which included healthNF and riskNF, as well as CAF with the S3 phenotype (Figure 1). A gradient was traced from this center toward PC1, and a distinct cluster of healthNFs with the S2 phenotype was located to the left and upward. Meanwhile, CAFs with the S1 and S4 phenotypes were located to the right and downward. The perpendicular direction (rightward and upward) was associated with increased CXCL12 expression (dot size on the graph), and was predominantly comprised of CAFs expressing the S1 phenotype, as well as isolated CAF-S4 and clustered CAF-S2. Furthermore, two samples of healthNFs with the S1 phenotype were identified in this region. A notable cluster of cancerNFs with the S3 phenotype was observed on the right side of the PCA plot, adjacent to CAF-S1. The spatial proximity of cancerNF-S3 and CAF-S1 indicated similarity in their transcriptome profiles, and the location of cancerNFs between healthNFs and CAFs corresponded to this group’s intermediate position in the healthNF → cancerNF → CAF trajectory.

**FIGURE 1:**
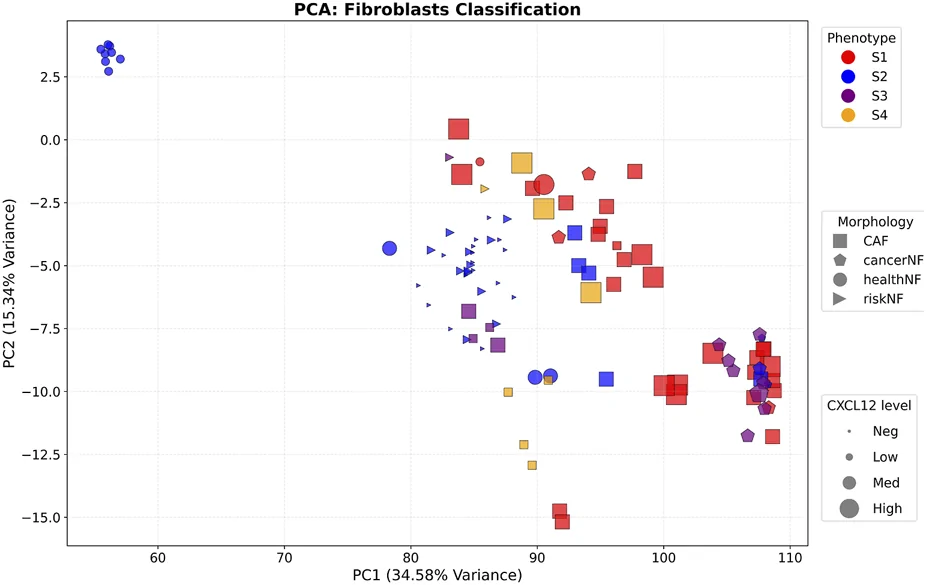
The phenotypic classification of mammary fibroblast isolates performed by integrating functional subtypes, morphology, and CXC chemokine ligand 12 (CXCL12) expression levels. Principal Component Analysis (PCA) was performed on a combined feature space comprising morphological parameters, phenotypic marker profiles, and normalized CXCL12 expression. Prior to analysis, the data were scaled to unit variance to ensure equal weighting of the heterogeneous data types. PC1 (34.58% variance) and PC2 (15.34% variance) are the two major orthogonal axes of variability. Percentages in parentheses indicate the proportion of total variance explained by each component. Each point in the graph represents an individual fibroblast isolate, with the total number of isolates used for the graph being 101. The color designations indicate the specific subtypes, designated as S1-S4. The distinction of sample morphology is predicated on the recognition of shape. The point size is proportional to relative CXCL12 expression level (higher expression = larger dots).

#### 3.2.2 Distribution of subtypes by clinical-morphological groups

A subsequent analysis of the contingency table “clinical-morphological group × subtype S1-S4” for the trajectory healthNF → cancerNF → CAF revealed a significant association (χ^2^, p = 6.85 × 10^−9^) (Supplementary Figure S2). Within the healthNF cohort, the S2 subtype was identified as the most prevalent (FDR = 2.23 × 10^−6^). At the cancerNF stage, the S3 subtype became dominant (FDR = 4.39 × 10^−5^). In the CAF group, a significant increase in the proportion of S1 (FDR = 6.46 × 10^−4^) was observed, while S2 (FDR = 6.46 × 10^−4^) and S3 (FDR = 4.98 × 10^−2^) were preserved, and the appearance of S4 (FDR = 4.98 × 10^−2^) was noted. The analysis of standardized residuals corroborated these patterns, with positive residuals observed for S2 among healthNF, S3 among cancerNF, and S1/S4 among CAF (Table 1).

**TABLE 1:** The distribution of subtypes S1–S4 by clinical and morphological groups for the *healthNF → cancerNF → CAF trajectory*. Absolute numbers and proportions are shown.

Group	CAF-S1	CAF-S2	CAF-S3	CAF-S4	Total
healthNF	2 (10,5%)	15 (79,0%)	2 (10,5%)	0 (0%)	19
cancerNF	3 (15,8%)	2 (10,5%)	14 (73,7%)	0 (0%)	19
CAF	28 (63,6%)	5 (11,4%)	4 (9,1%)	7 (15,9%)	44
Total	33	22	20	7	82

For the alternative trajectory healthNF → riskNF → CAF, significant associations (χ^2^, p = 2.72 × 10^−10^) were also identified. In the healthNF stage, S2 was found to be the predominant form (FDR = 1.87 × 10^−2^) (Supplementary Figure S3). Among riskNF, the dominance of S2 was confirmed (FDR = 2.86 × 10^−8^). Upon transition to CAFs, a significant increase in the proportion of S1 was observed (FDR = 2.86 × 10^−8^), accompanied by a decrease in the proportion of S2 (FDR = 1.64 × 10^−12^). The absence of S1 samples with a significant FDR (2.74 × 10^−6^) at the riskNF stage and insignificant residuals for S3 and S4 at this stage indicated that the observed changes in distribution were not random (Table 2).

**TABLE 2:** The distribution of subtypes S1–S4 by clinical and morphological groups for the *healthNF → riskNF → CAF* trajectory. The absolute numbers and proportions are displayed.

Group	CAF-S1	CAF-S2	CAF-S3	CAF-S4	Total
healthNF	2 (10,5%)	15 (79,0%)	2 (10,5%)	0 (0%)	19
riskNF	0 (0%)	19 (100%)	0 (0%)	0 (0%)	19
CAF	28 (63,6%)	5 (11,4%)	4 (9,1%)	7 (15,9%)	44
Total	30	39	6	7	82

### 3.3 Co-expression patterns of CAF and CXCL12 marker genes

#### 3.3.1 Correlations across the entire dataset

A thorough investigation into the correlation matrix derived from 101 samples revealed a predominantly positive correlation between the panel genes (Figure 2). The sole exception was CAV1, which exhibited a substantial negative correlation with the majority of the markers: FAP (r = −0.55), CXCR4 (r = −0.52), ACTA2 (r = −0.45), CXCL12 (r = −0.38), and PDGFRB (r = −0.33). The highest positive correlation was observed between TGFB1 and ITGB1 (r = 0.72), as well as between ITGB1 and FAP (r = 0.71). CXCL12 was characterized by the largest number of strong positive correlations with markers of the CAF phenotype: The following genes were found to be significantly correlated: ACTA2 (r = 0.69), FAP (r = 0.67), ITGB1 (r = 0.64), TGFB1 (r = 0.63), PDGFRB (r = 0.42), and S100A4 (r = 0.34). However, no significant correlation was found between the chemokine CXCL12 and its receptor, CXCR4. An analysis of the CXCL12/CXCR4 scatterplot revealed that CXCR4 expression exhibited significant variability across the sample. In the majority of samples, CXCR4 expression was minimal, which limited the ability to detect this relationship using bulk RNA-seq.

**FIGURE 2:**
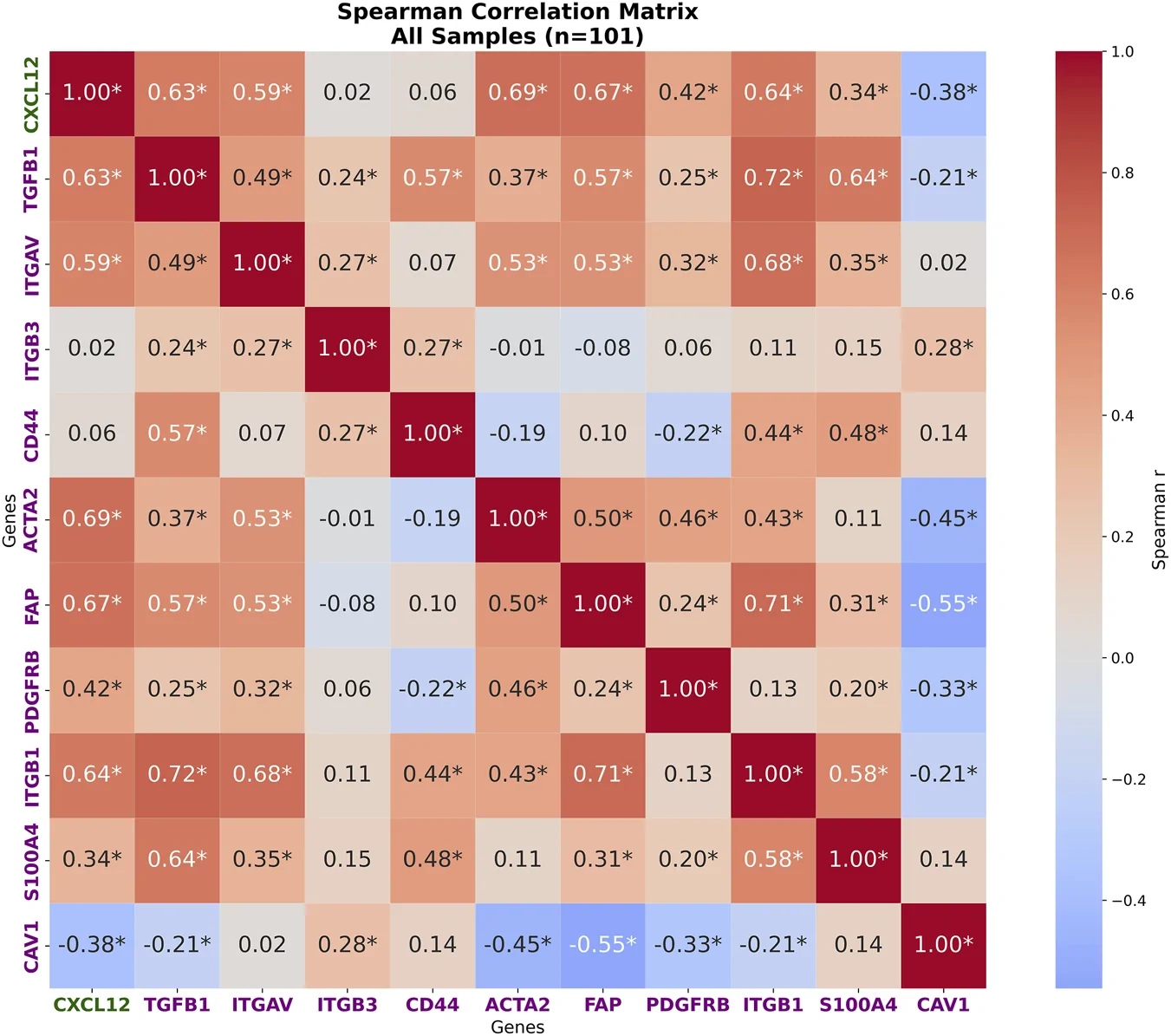
Correlation matrix between CXCL12 expression and breast fibroblast marker genes among all samples.

Subsequent experimentation was thus initiated in order to evaluate the validity of the hypothesis that the autocrine loop involving the chemokine, CXCR4, is indeed valid. This objective was realized through the comparison of samples that exhibited the highest CXCR4 expression levels (designated as “High”) with the remaining samples. Furthermore, the role of CXCR4 in synergy with TGF-β was analyzed, with receptor expression being excluded from consideration. Scatterplots for all gene pairs in the panel are provided in Supplementary Figure S4.

#### 3.3.2 Correlations across the clinical-morphological groups

The construction of correlation matrices, with each matrix created separately for each clinical and morphological condition, revealed significant differences in co-expression patterns (Supplementary Figure S5). In the healthNF group, strong positive correlations were observed between most markers: ITGB1 with CAV1, TGFB1 with ITGB3 and CD44, as well as TGFB1 with ITGB1, CAV1, and ACTA2. In the cancerNF group, a change in the sign of several correlations was observed. The FAP-CAV1 correlation became sharply negative, CAV1 changed its correlation sign with ACTA2 and ITGAV, TGFB1 with FAP changed from positive to negative, and CXCL12 with ITGB1 changed from positive to negative. In the CAF group, the coordination of gene expression virtually disappeared, with only a strong negative correlation remaining between ACTA2 and CD44.

The transition from healthNF to riskNF resulted in a weakening of the correlations between genes, while the correlations between CXCL12 and CD44 became moderately negative (Supplementary Figure S5). Stable positive correlations were maintained between ITGAV and ITGB3, ACTA2, and FAP; moreover, the ITGAV-PDGFRB correlation strengthened. In addition, PDGFRB demonstrated novel positive correlations with multiple other factors, including CXCL12, ITGB3, ITGB1, and FAP. Upon transitioning to CAF, these correlations also almost completely disappeared in this trajectory.

### 3.4 Differential expression of genes across the clinical-morphological groups

A comparison of the healthNF group with the combined riskNF, cancerNF, and CAF group revealed significant differences in the expression of TGFB1 and a number of CAF markers (ITGB1, S100A4, CAV1) (Figure 3). However, this combined comparison does not allow for the differentiation of changes specific to individual stages of the healthNF → cancerNF or cancerNF → CAF transformation.

**FIGURE 3:**
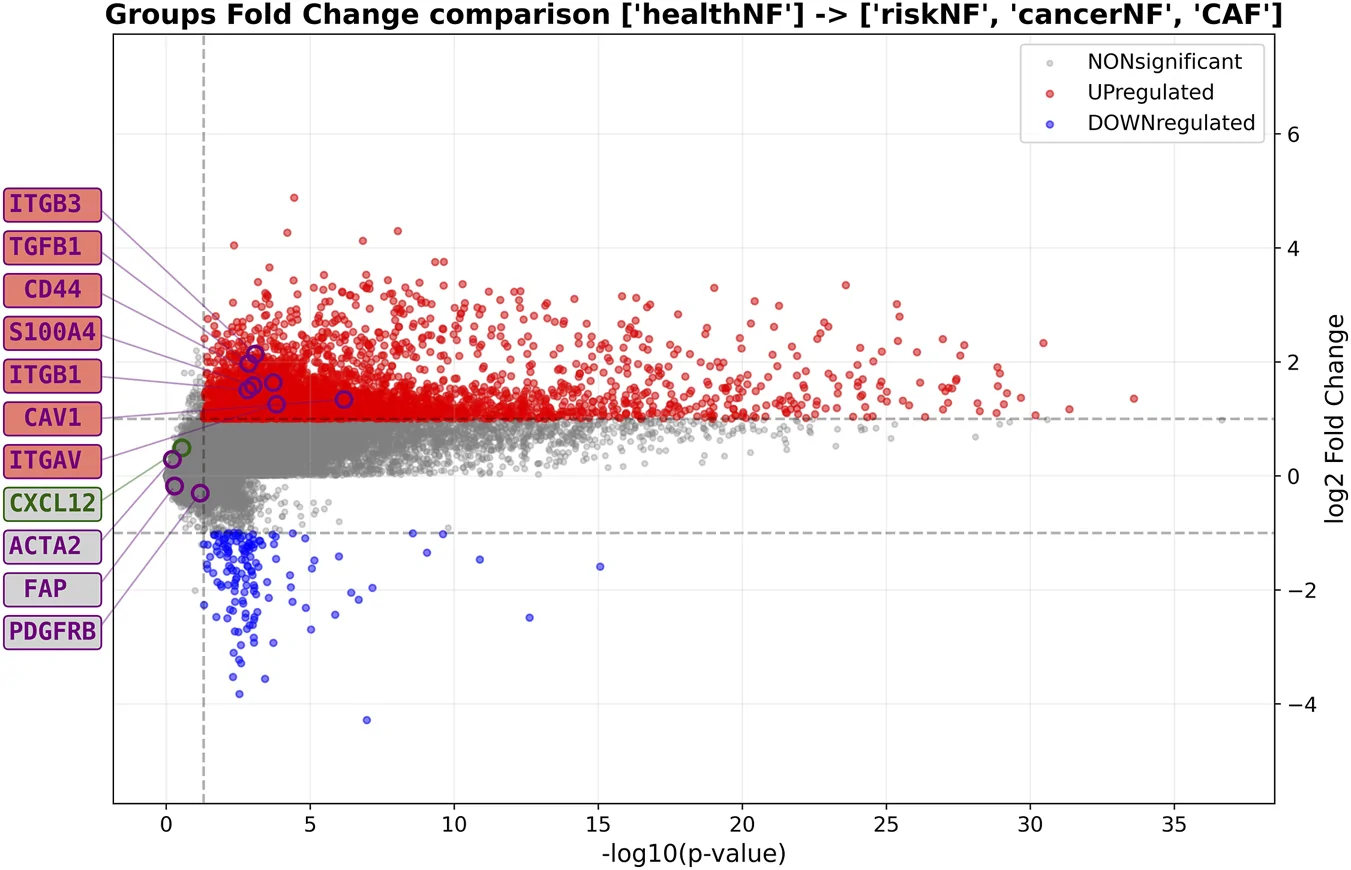
Differential expression of CXCL12 and breast fibroblast marker genes in the combined riskNF + cancerNF + CAF group compared to the healthNF group.

A separate DE analysis for each pair of conditions demonstrated that the number of upregulated genes in riskNF was significantly higher (2,887) than in CAF (724) and cancerNF (540) (Figure 4). CXCL12 and CAF markers (ITGB1, S100A4, CAV1) exhibited a marked increase in expression during the transition from healthNF to CAF, as well as at intermediate stages of riskNF and cancerNF. In contrast, the expression levels of FAP and PDGFRB decreased in the riskNF group, distinguishing this group from cancerNF and CAF. The divergent nature of the alterations (increased CXCL12 in cancerNF and CAF, increased ACTA2 only in CAF, and decreased expression of both in riskNF) is consistent with the existence of two groups of trajectories: healthNF → cancerNF → CAF and healthNF → riskNF → CAF. These trajectories were analyzed separately in subsequent sections.

**FIGURE 4:**
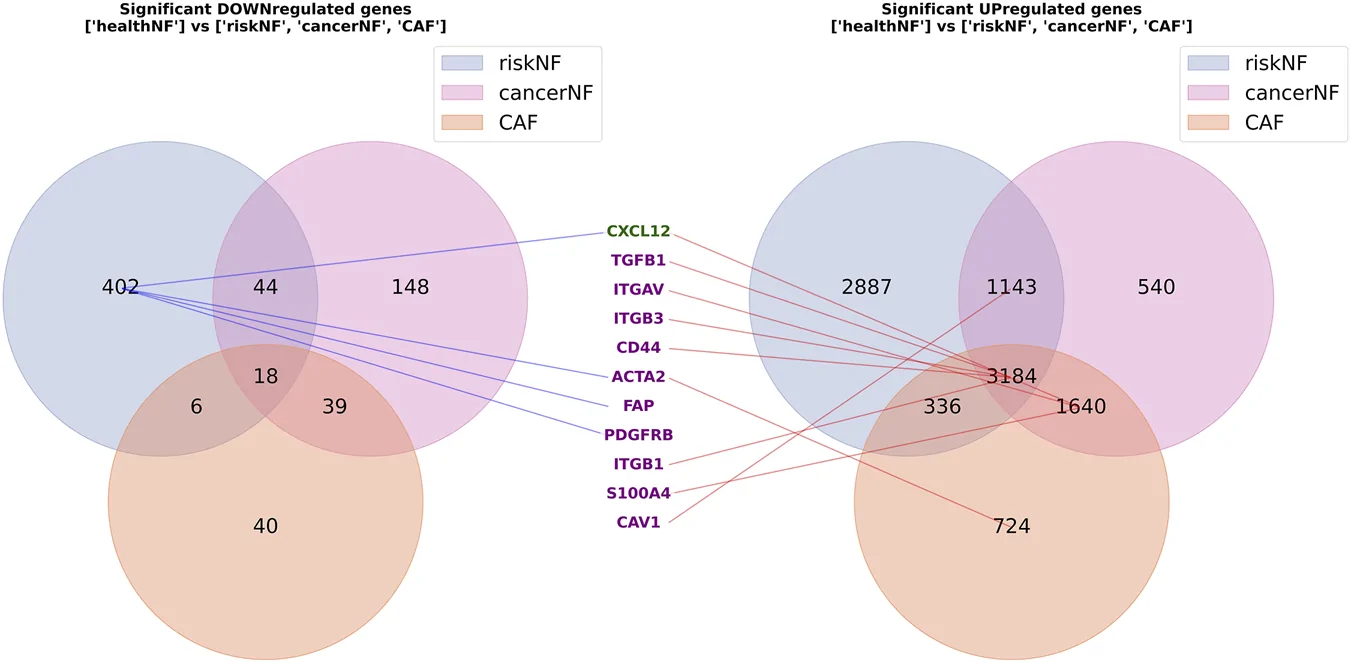
Differential expression of CXCL12 and marker genes between the healthNF group and combined riskNF + cancerNF + CAF breast fibroblast group separately with counting of total down-regulated and up-regulated genes.

### 3.5 Differential expression across trajectories and fibroblast subtypes

A DE analysis in fibroblast groups along the healthNF → cancerNF → CAF trajectory revealed a significant increase in the expression of the main part of the marker protein genes characteristic of activated fibroblasts, including S100A4, ITGB1, and CAV1 (Supplementary Figure S6). A detailed examination of the DE analysis, categorized according to fibroblast subtypes, revealed that at the cancerNF stage, an increase in TGFB1 is a distinguishing feature of all the presented subtypes. In contrast, the expression of CXCL12 is exclusively observed in S1 and S3 subtypes (Figure 5).

**FIGURE 5:**
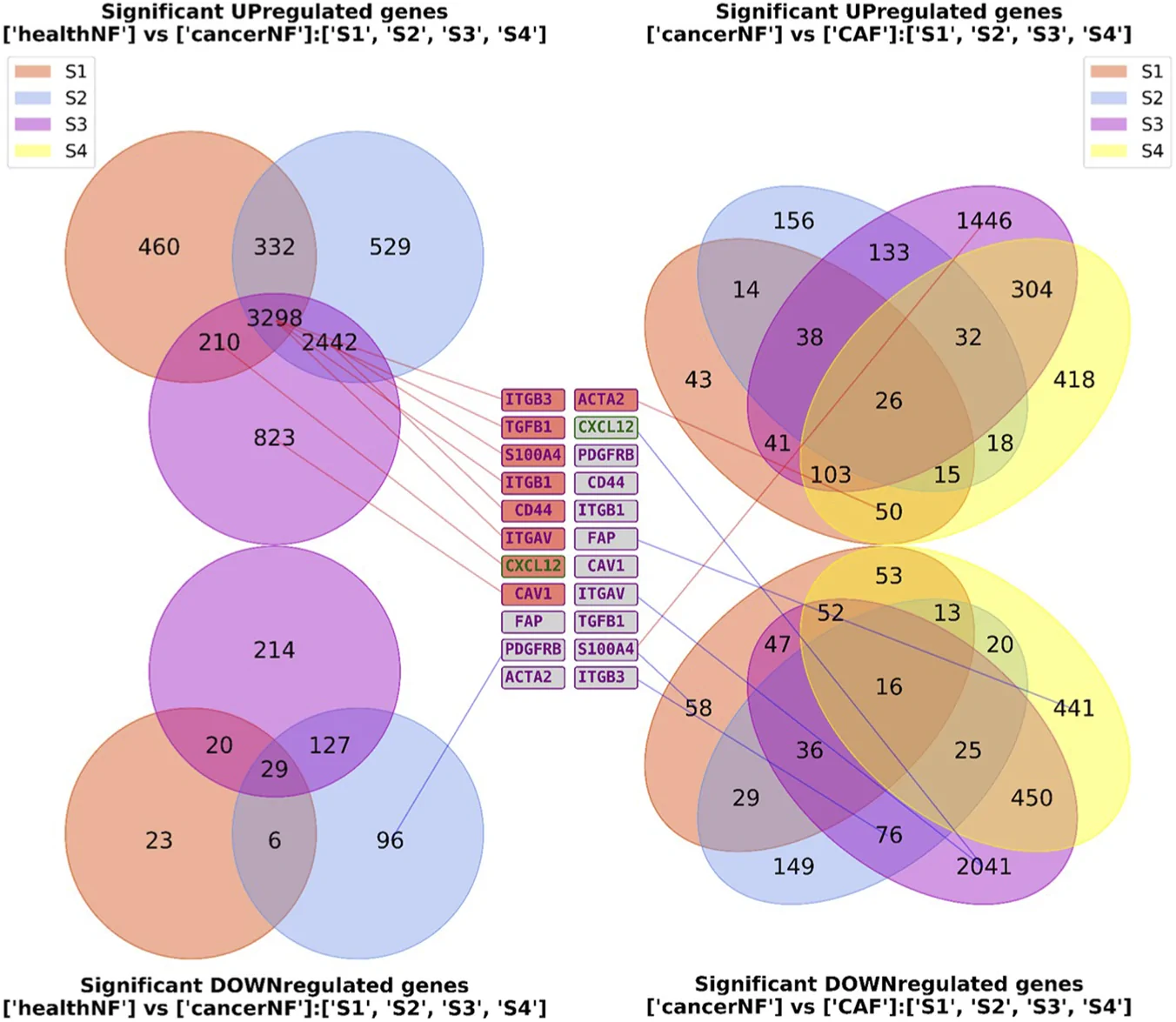
Description of differentially expressed genes in different fibroblast subsets along the healthNF → cancerNF → CAF trajectory.

The ITGAV and ITGB3 receptors demonstrated significant upregulation in all subtypes at the cancerNF stage, while CD44 exhibited upregulation in S2 and S3. Among the activated fibroblast markers, ITGB1 exhibited an increase in expression across all phenotypes, while S100A4 demonstrated an increase in expression in S2 and S3, and CAV1 exhibited an increase in expression exclusively in S3. No samples exhibiting the S4 phenotype were identified during the cancerNF stage of the study. Subtype S3 was distinguished by the most significant number of upregulated genes (822) in comparison to S1 (460) and S2 (529). A total of 3,298 common upregulated genes were identified across all subtypes at the cancerNF stage. The transition from cancerNF to CAF revealed a significant reduction in the number of common genes, with the total dropping to 26. Of the panel markers, ACTA2 exhibited significant upregulation in S1 and S4, while S100A4 demonstrated upregulation in S3. Conversely, FAP showed a decrease in S4.

In the healthNF → riskNF → CAF trajectory, the riskNF stage significantly increased the expression of CAV1, ITGB3, and CD44 (Supplementary Figure S7). TGFB1 and ITGB1 exhibited a moderate yet statistically significant increase. In contrast, the expression of CXCR4 demonstrated the most pronounced decrease, followed by ACTA2, FAP, and PDGFRB. S100A4 and ITGAV exhibited no substantial alterations. In contrast, during the transition from riskNF to CAF, the direction of changes was antithetical. Specifically, genes that exhibited decreased expression at the riskNF stage (CXCL12, ACTA2, FAP, PDGFRB) significantly increased their expression at the CAF stage, following the same relative order. CAV1, on the other hand, exhibited a transition from maximal increase to maximal decrease. TGFB1, S100A4, ITGB1, CD44, ITGAV, and ITGB3, activated at the risk NF stage, maintained their achieved levels without significant changes. A thorough investigation into fibroblast subtypes was conducted via DE analysis, which revealed that the number of upregulated genes at the risk NF stage (phenotype S2) was 7,559, while the number of downregulated genes was 512 (Figure 6). During the transition to CAF, a total of 854 genes were found to be upregulated, while 866 genes were downregulated. CXCL12 demonstrated an increase in all CAF subtypes, while TGFB1 exhibited a selective increase in S1. S100A4 levels exhibited an increase in S2 and S3. CAV1 expression was found to be decreased in all observed phenotypes.

**FIGURE 6:**
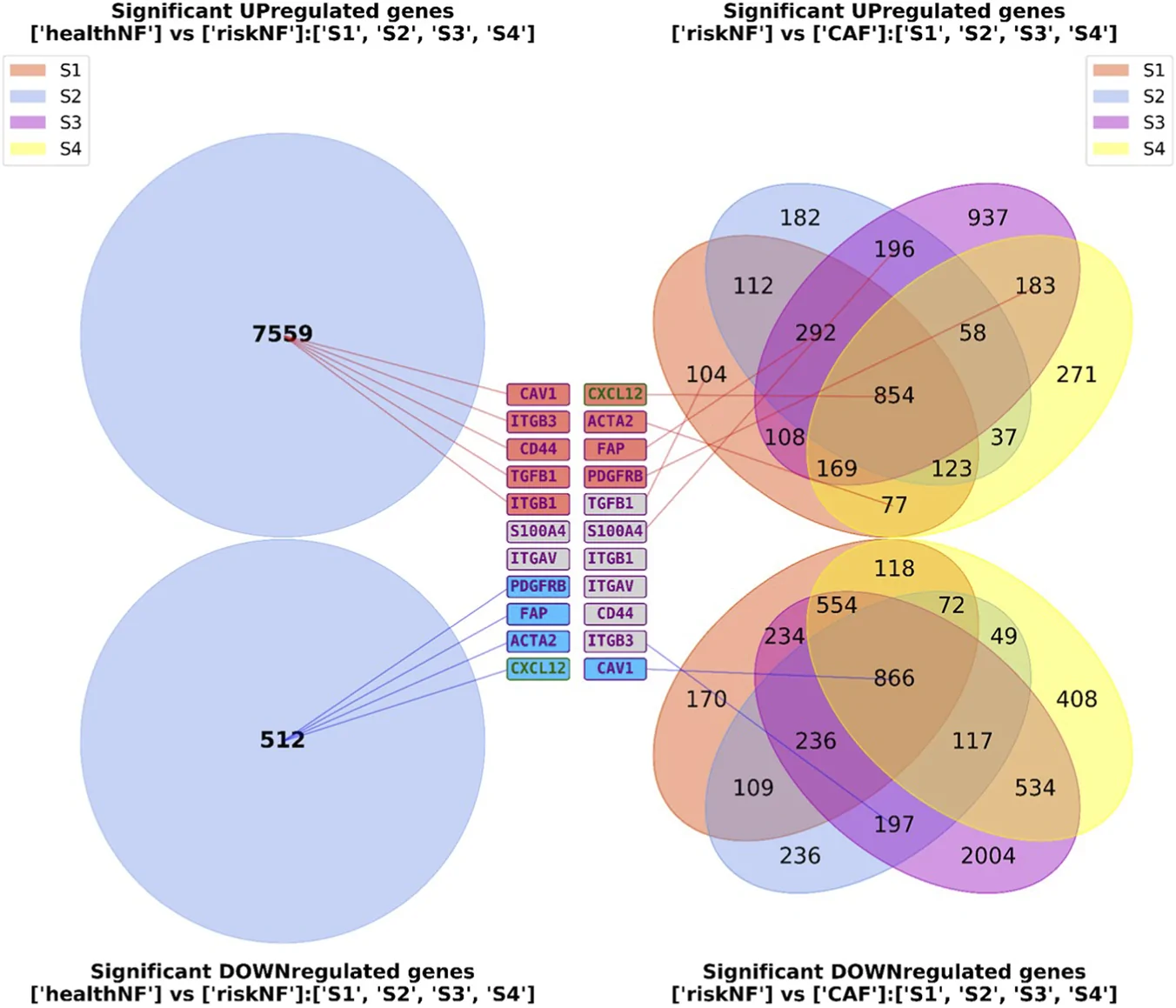
Description of differentially expressed genes in different fibroblast subsets along the healthNF → riskNF → CAF trajectory.

### 3.6 Effect of CXCR4 expression on the CXCL12/CXCR4 axis and the synergy with TGF-β

#### 3.6.1 Distribution of CXCR4 expression levels

The distribution of CXCR4 expression levels (*Neg, Low, Med, High*) across clinical and morphological groups was analyzed, revealing that the High level was exclusively observed in the CAF group (10 samples; FDR = 5.98 × 10^−4^). In the healthNF, riskNF, and cancerNF groups, CXCR4 expression did not reach a high level. The amalgamation of all non-CAF groups (healthNF, riskNF and cancerNF) into a single category resulted in a statistically significant absence of samples with a high level (Supplementary Figure S8). A thorough analysis of standardized residuals confirmed a significant excess of CAFs with high CXCR4 levels (residual = 2.70; observed = 10; expected = 4.4), while concurrently indicating a deficit within the combined non-CAF group.

A comparative analysis of the distribution of CXCR4 expression levels within designated groups was conducted. This analysis revealed that, at the CAF stage, there was a decline in the proportion of samples exhibiting *Neg* levels from 52.6% (combined healthNF, riskNF, cancerNF group) to 40.9%. Additionally, there was a decrease in the proportion of *Low* levels from 38.6% to 20.5%, while the proportion of *Med* levels exhibited an increase from 8.8% to 15.9%, and the proportion of *High* levels increased from 0% to 22.7% (Supplementary Figure S9).

When the CAFs were stratified by S1-S4 phenotypes, a sharp increase in the proportion of S1 (from 8.8% to 63.6%) was observed at the CAF stage, owing to a decrease in the proportion of S2 (from 73.7% to 11.4%). The proportion of S3 decreased marginally (from 15.8% to 9.1%), while the proportion of S4 increased (from 1.8% to 15.9%). The phenotype × CXCR4 level contingency table was analyzed, revealing that at *Neg, Low*, and *Med* levels, the numerical predominance of S2 remained (45.8%, 51.6%, and 75.0%, respectively), with a concomitant proportion of S1 (33.3% for *Neg*, 38.7% for *Low*) (Supplementary Figure S10). Upon reaching the *High* level, which was exclusively observed in CAF, a significant shift in the distribution was noted, with a notable increase in the proportion of S3 to 40.0% (FDR = 3.71 × 10^−2^) and S4 to 40.0% (FDR = 1.21 × 10^−3^). Subsequent narrowing of the analysis to CAF, excluding the remaining groups, did not yield statistically significant results due to the limited number of observations in most categories, at which point the chi-square test becomes ineffective.

#### 3.6.2 Correlations of CXCL12 and TGFB1 at different CXCR4 levels

In the complete absence of CXCR4 expression (*Neg* level), a high positive correlation of CXCL12 with TGFB1 and differentiation markers (ACTA2, FAP, PDGFRB, ITGB1) was observed (Figure 7). CXCL12 exhibited a more robust correlation with ACTA2 and PDGFRB than TGFB1, while TGFB1 demonstrated a stronger correlation with S100A4. Furthermore, a moderate negative correlation was identified between CXCL12 and CAV1. ACTA2, FAP, PDGFRB, and ITGB1 exhibited a moderate correlation with each other. When CXCR4 expression manifested at *Low* and *Med* levels, the previous correlation between the markers was found to be diminished. The correlations between PDGFRB and the remaining genes were no longer statistically significant. The negative correlations of CAV1 exhibited a decline. Conversely, S100A4 exhibited a strengthening of the correlation with both CXCL12 and TGFB1. The correlation between CXCL12 and TGFB1 exhibited its most significant association at these specific levels of CXCR4. Upon attaining a high level of CXCR4, the correlation between CXCL12 and TGFB1 became negative. TGFB1 altered its correlation with ITGAV, rendering it strongly negative, while acquiring a robust positive correlation with CD44. The correlations between TGFB1 and FAP, as well as TGFB1 and ITGB1, exhibited a negative trend. Conversely, CXCL12 exhibited a positive correlation with ITGAV, further enhancing its association with FAP and ITGB1, approaching near-functional levels. FAP and ITGB1 exhibited a strong correlation with each other and with ITGAV.

**FIGURE 7:**
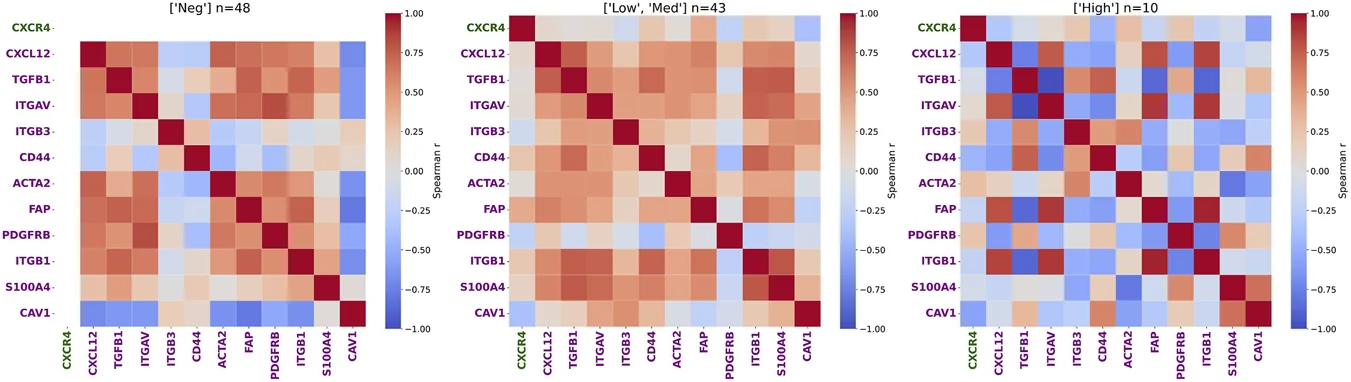
Correlation matrices of gene expression at different levels of CXCR4 expression.

#### 3.6.3 Differential expression at stratification by CXCR4

When groups with different CXCR4 levels were compared without taking into account phenotype, no significant changes in gene expression were detected. However, upon disaggregation of the data according to subtype, a significant increase in the expression of the chemokine, CXCR12, was observed in the S1 subtype, at both the low and medium levels of the CXCR4 protein (Figure 8). In this subtype, ACTA2 expression exhibited a marked increase. In the S2 subtype, with equivalent CXCR4 levels, ACTA2 and ITGB3 expression was found to be significantly diminished. Among the samples exhibiting high CXCR4 levels, the S2 subtype was not detected. In the S4 subtype, a decrease in FAP with an increase in PDGFRB was observed, as well as an increase in ITGB3 in S4 and S1. The presence of elevated CXCR4 levels was indicative of the S1, S3, and S4 subtypes, and was exclusively detected in the CAF group.

**FIGURE 8:**
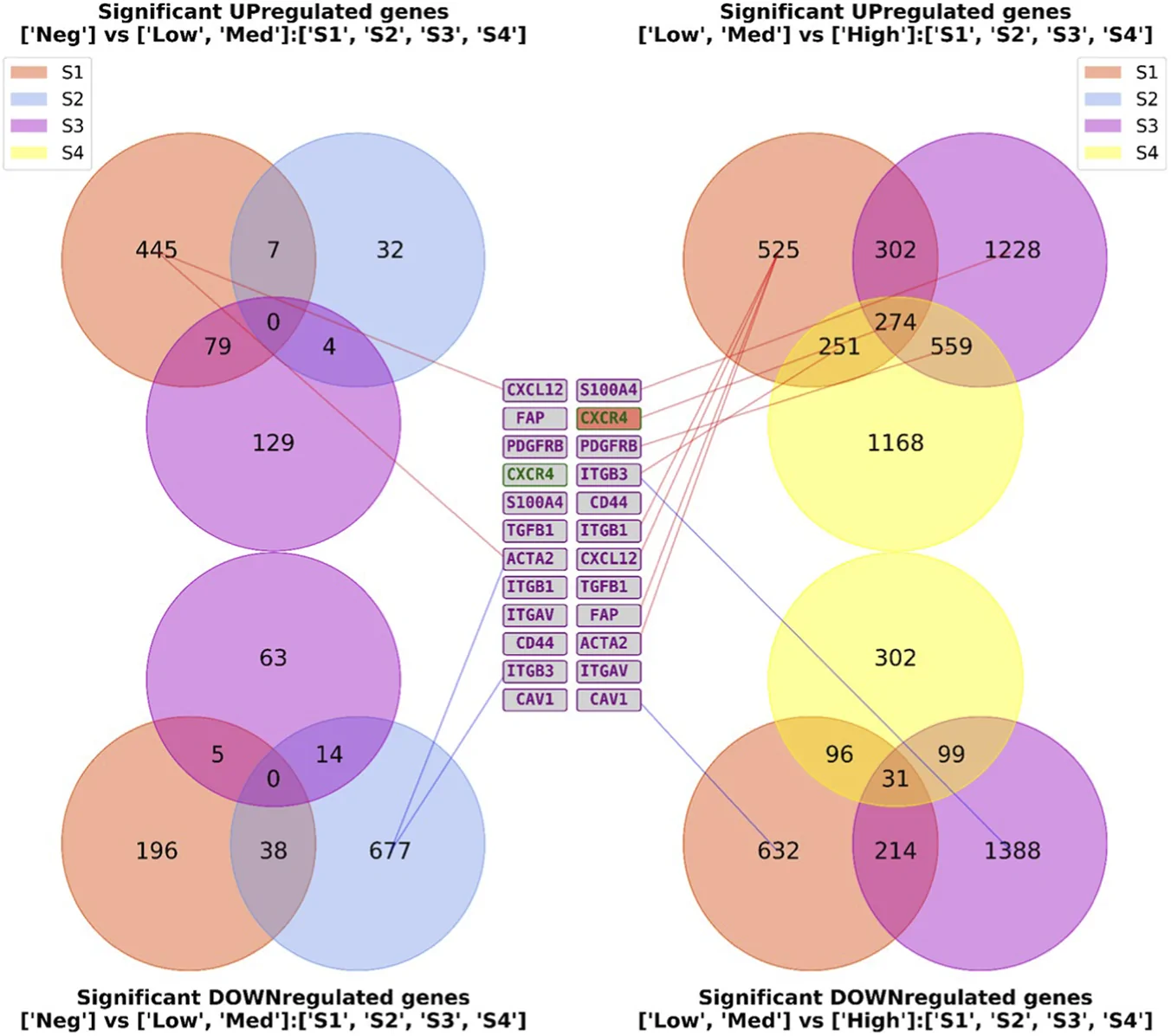
Description of differentially expressed genes in various subsets of CXCR4-expressing fibroblasts.

### 3.7 GSEA: biological processes in CAFs with high CXCR4 levels

A Gene Set Enrichment Analysis (GSEA) was performed to compare CAFs with CXCR4^High^ levels against CAFs with *Neg*, *Low*, and *Med* levels. This analysis revealed significant enrichment (FDR <0.25) of gene sets in biological process and pathway signatures from the MSigDB Hallmark 2020 collection, separating into opposite patterns (Figure 9). In the CXCR4^High^ group, the following gene sets associated with the immune and inflammatory response were found to be significantly enriched (NES >0): Interferon gamma response, Interferon alpha response, TNF-alpha signaling via NF-kB, Inflammatory response, IL-6/JAK/STAT3 signaling, Allograft rejection, the p53 pathway, KRAS signaling up, and Apoptosis. Concurrently, within the CXCR4-*High* cohort, the enrichment of sets associated with proliferation and the cell cycle was markedly diminished (NES <0): G2-M Checkpoint, E2F Targets, Myc Targets V1 and V2, Mitotic Spindle, as well as Epithelial Mesenchymal Transition, TGF-beta Signaling, Unfolded Protein Response, Angiogenesis, Glycolysis, and mTORC1 Signaling sets. Consequently, the transcriptome profile of CAFs with high CXCR4 levels is marked by the activation of immunomodulatory and pro-inflammatory programs, accompanied by a concurrent decrease in proliferative, myofibroblastic, and metabolic activity.

**FIGURE 9:**
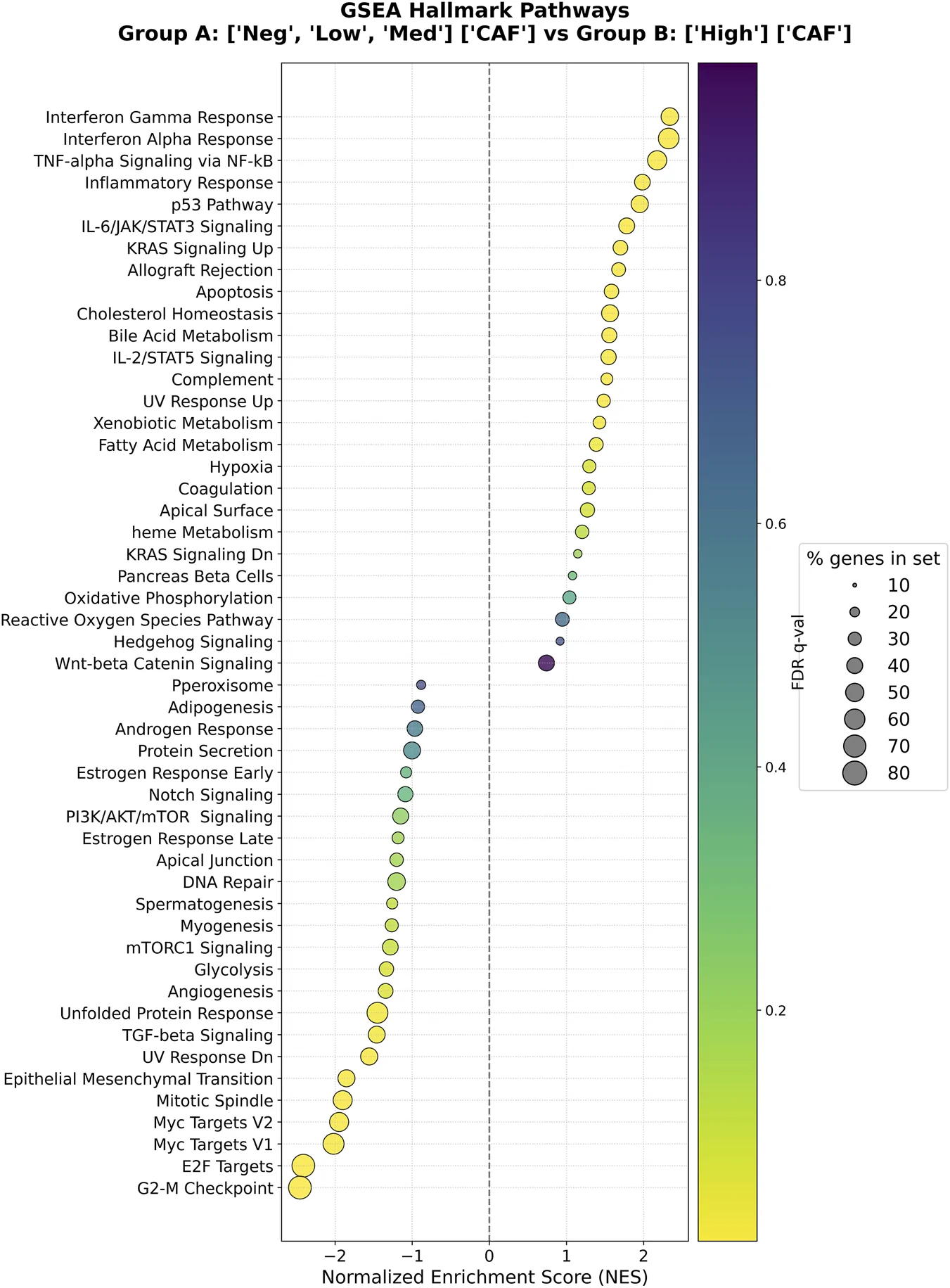
Description of enrichment analysis of expressed genes in different subsets of CXCR4-expressing fibroblasts using Normalized Enrichment Scores (NES).

## 4 Discussion

The present study set a goal to investigate the association between high CXCR4 expression in mammary gland CAFs and altered gene co-expression patterns. The hypothesis was that these patterns would be consistent with a switch from paracrine (CXCL12 → CXCR4 of tumor cells) to autocrine (CXCL12 → CXCR4 of the same CAFs) regulation of SDF-1α. Nevertheless, this was regarded as a working hypothesis rather than a mechanism demonstrated by bulk RNA-seq. A subsequent analysis of the STRING database revealed that TGFB1 is annotated as a participant in epithelial-mesenchymal transition processes (GO:0001837), fibroblast proliferation regulation (GO:0048145), and positive regulation of their migration (GO:0010763). In contrast, analysis of the CXCR4 database indicated that the function of the (CXCL12) is predominantly associated with the development of blood vessels (GO:0001525), realized through CXCR4 on endothelial progenitor cells, and positive regulation of cell differentiation (GO:0045597) (Figure 10). It is noteworthy that STRING reflects known literature data, rather than expression patterns in the fibroblast sample set studied. The database contains strong associations of TGFB1 with ITGB1, PDGFRB, and ACTA2, which correspond to experimentally established interactions. The expression correlations that were analyzed reflect co-regulation at the mRNA level in specific clinical groups. A comparison of these two sources of information enables the interpretation of the obtained correlations within the context of known molecular interactions, without conflating them with evidence for specific mechanisms.

**FIGURE 10:**
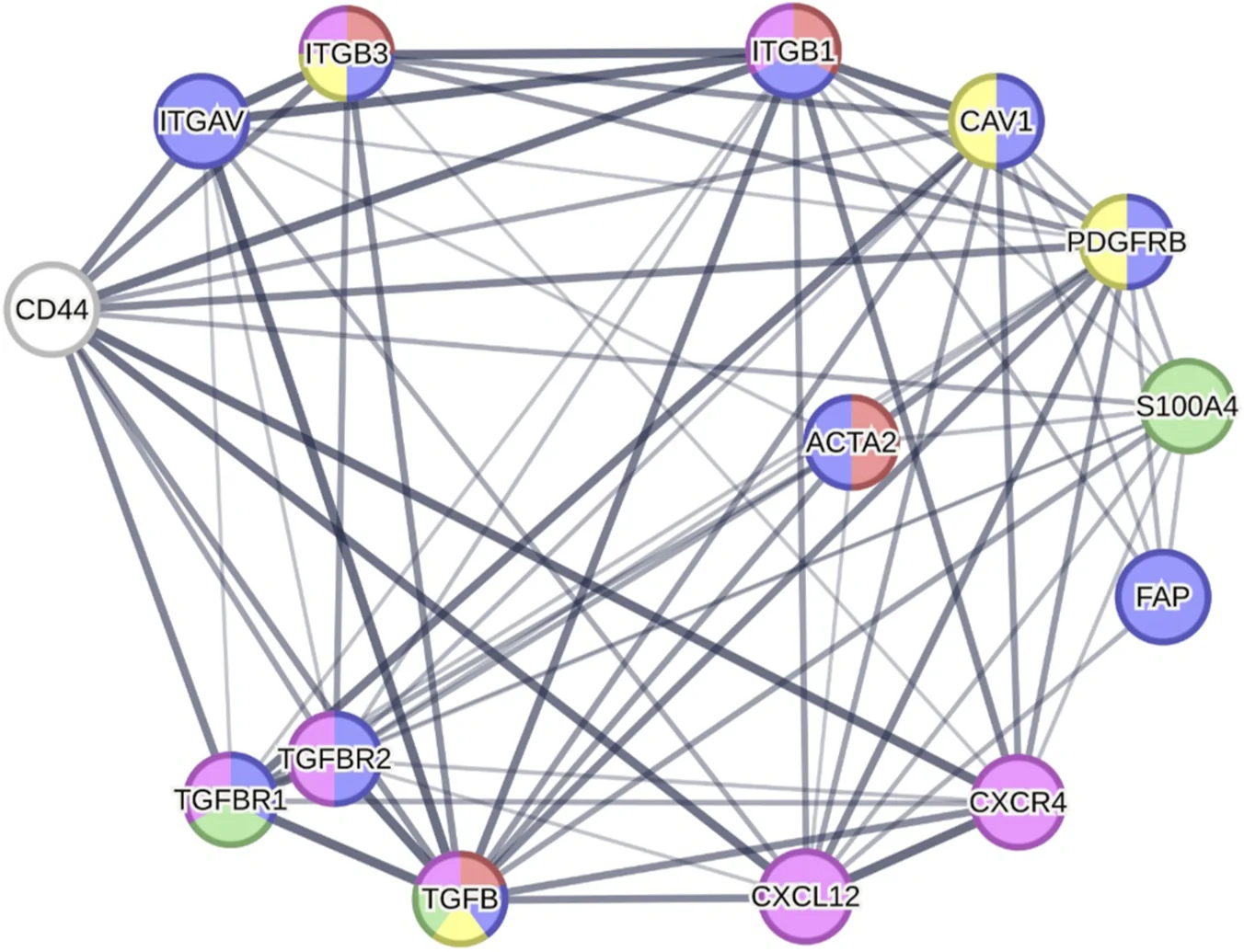
Protein-protein interaction network of the studied targets according to the STRING database. Green (GO:0001837) - Epithelial to mesenchymal transition; Yellow (GO:0048145) - Regulation of fibroblast proliferation; Red (GO:0010763) - Positive regulation of fibroblast migration; Blue (GO:0001525) - Blood vessel development; Pink (GO:0045597) - Positive regulation of cell differentiation. https://string-db.org/cgi/network?taskId=bBldYsPc7Z27&sessionId=b4XOuC2CX4iq. The network comprises 14 nodes and 71 edges (average node degree = 10.1, average local clustering coefficient = 0.846). The network has significantly more interactions than expected (expected number of edges = 10, PPI enrichment p value <1.0e^−16^). The excess of internal connections compared to a random protein set of comparable size indicates that this network is non-random and reflects genuine functional coupling among its members.

In the studied set of fibroblast samples, in the absence of CXCR4 expression (*Neg* level), a positive correlation of CXCL12 with CD44 was observed. CD44 was identified as a receptor for osteopontin (OPN), also known as secreted phosphoprotein 1 (SPP1), and hyaluronic acid (Panda et al., 2024; Vasilevska et al., 2024).

OPN is increasingly recognized as a central mediator within the TME, where it may help coordinate cellular interactions, immune evasion, and therapeutic resistance. It has been demonstrated that this process facilitates bidirectional communication between cancer cells and key stromal components, including CAFs, TAMs, endothelial cells, and lymphocytes. This, in turn, supports the organization of complex cellular networks (Panda et al., 2024; Wang L. et al., 2026b; Zhao et al., 2024; Zheng et al., 2025). Butti et al. (2021) showed that tumor-derived OPN promotes the differentiation of resident fibroblasts into myofibroblasts through activation of the transcription factor Twist1, which regulates the expression of canonical CAF markers, including FAP, α-SMA, FSP1, CD29, and PDGFRβ. OPN was also reported to induce SDF-1 expression, thereby providing a potential mechanistic link between OPN-mediated fibroblast activation and CXCL12 (SDF-1) – TGF-β signaling crosstalk (Louault et al., 2020; Wu et al., 2021; Butti et al., 2021; Gu et al., 2024).

More recently, SPP1 has emerged as a pivotal regulator of stromal fibroblasts and immune cells across diverse contexts. In the study by Huang et al. (2025), the intricate cellular interactions between MMP11^+^-CAFs and SPP1^+^-TAMs were meticulously delineated. This seminal research underscores the significance of these factors as prospective therapeutic targets for future BC treatments (Huang et al., 2026). Transcriptomic studies of skeletal muscle have identified an association between SPP1^+^ macrophages and fibrotic tissue remodeling, indicating that SPP1-associated immune–mesenchymal crosstalk is not restricted to tumors (Palma, 2025). Vitaliti et al. (2024) integrated single-cell datasets from dystrophic skeletal muscle and described coordinated macrophage and fibro-adipogenic progenitor profiles (Vitality et al., 2024). More broadly, Reggio et al. (2025) have associated SPP1^+^ macrophages with ECM remodeling, fibrosis, and immunomodulation in aging, muscle disease, and cancer (Reggio et al., 2025).

The co-expression of CD44 with CXCL12 in the absence of CXCR4 is consistent with several hypotheses. Firstly, both genes are known to be activated by a shared transcriptional regulator in the context of quiescent fibroblasts. Secondly, CXCL12 has been observed to function through an alternative receptor ACKR3/CXCR7, which is not included in the study (Koch et al., 2020; Yang Y. et al., 2023). However, the obtained data do not allow for the distinction between these variants.

The role of CXCL12 in tandem with CXCR4, is evident in the regulation of cellular differentiation (GO:0045597). The correlations of CXCL12 with FAP phenotype markers in the studied sample are weaker than those of TGFB1, which is consistent with the concept of TGF-β as the primary driver of myofibroblast differentiation at the early stages of fibroblast activation (Webber et al., 2009; Chen et al., 2025; Wang et al., 2025).

It should be noted that the integration of transcriptomic data from multiple independent studies may be accompanied by technical and biological variability arising from differences in library preparation protocols, sequencing platforms, sequencing depth, annotation methods, and primary data processing conditions. Despite the use of uniform selection criteria and a unified analytical pipeline, the influence of batch effects cannot be entirely excluded. Therefore, the results obtained should be interpreted as reflecting robust associative patterns rather than as definitive proof of a biological transition between states.

### 4.1 The two trajectories of fibroblast activation

The sequence of CAF transformation and carcinogenesis in the mammary gland remains unclear. The hypothesis posits that the reprogramming of normal fibroblasts into CAFs may occur prior to the onset of carcinogenesis. CAFs have also been observed to induce the transformation of nearby epithelial cells into more malignant cells prior to a cumulative surge of mutations and genetic alterations in normal mammary epithelial cells (Zhou et al., 2025; Dumont et al., 2013; Kuperwasser et al., 2004). In the event of the mutation of multiple oncogenes in normal mammary epithelial cells, their proliferation becomes unregulated by standard physiological functions. This phenomenon is associated with an increased risk of developing BC, a condition marked by uncontrolled cell growth. At the precancerous stage, the presence of BRCA1^+/mut^ fibroblasts was observed to exhibit a CAF phenotype, characterized by elevated expression of genes associated with CAFs and inflammation, in comparison to individuals who were mutation-negative (Nee et al., 2023). *In vivo*, the presence of fibroblasts expressing matrix metalloproteinase-3 has been observed to stimulate the development of BC harboring a BRCA1 mutation via a paracrine pathway (Wooster et al., 2003).

The results of PCA and contingency table analysis enabled the description of two patterns of CAF subtype distribution across clinical and morphological groups. In the healthNF → cancerNF → CAF trajectory groups, the S2 (healthNF group, 79.0%), S3 (cancerNF group, 73.7%), and S1/S4 (CAF group, 63.6% and 15.9%, respectively) subtypes consistently predominated. In the healthNF → riskNF → CAF trajectory groups, the S2 subtype exhibited a predominant presence in both healthNF (79.0%) and riskNF (100%), while in CAF, the S1 share was 63.6%, indicating a substantial decline in the S2 share to 11.4%. It is imperative to elucidate that the data were obtained through a cross-sectional study design. In this design, each group represents independent samples, rather than the same cells being tracked over an extended period.

It is imperative to emphasize that, in this study, the designations S1-S4 were derived from bulk RNA-seq data using the same combinations of markers that had previously been identified at the single-cell and single-protein levels. Consequently, the transcriptomic clusters represented by the CAF-S1, CAF-S2, CAF-S3 and CAF-S4 groups are enriched for the corresponding marker expression patterns. However, these clusters do not necessarily correlate exactly with the functional CAF subtypes described in studies using imaging or flow cytometry. Consequently, the observed enrichment of S1- and S4-like signatures in various clinical-morphological groups should be interpreted as an indication of relative shifts in the spectrum of fibroblast activation states, rather than as direct evidence of an expansion or contraction of specific, functionally verified CAF lineages.

CAF-S1 (ACTA2^high^, FAP^high^, PDGFRβ^med/high^, CAV1^low^) is a myofibroblast subtype associated with immunosuppression (Thiery, 2022). The immunosuppression mechanism involves the secretion of TGF-β1, which suppresses the cytotoxic activity of CD8^+^ T cells by reducing the expression of perforin and granzyme B. It also involves the secretion of CXCL12, which retains T cells in the perivascular niche and prevents their penetration into the tumor compartment. Finally, it involves extracellular matrix remodeling via FAP-dependent collagen degradation (Costa et al., 2018; Pelon et al., 2020; Chen et al., 2021).

CAF-S4 (ACTA2^high^, FAP^low/neg^, ITGB1^high^, PDGFRβ^low^) are enriched in genes regulating the actin cytoskeleton and oxidative metabolism (Erdogan et al., 2017; Li et al., 2021). It has been demonstrated that CAF-S4 possesses the potential to transdifferentiate into CAF-S1 (Xia et al., 2025). It has been posited that TGF-β1, acting through TGFBR2/ALK5 → SMAD3, induces FAP transcription. While the maintenance of survival and the myofibroblast phenotype is achieved by CXCL12, and is accomplished through the action of CXCR4 → Gi → PI3K/AKT axis (Chung et al., 2021; Walton et al., 2017). A specific transcription factor that triggers FAP expression during this transition has not been identified in the literature (Antonova et al., 2023; Cheng, 2010; Krepela et al., 2021). CAF-S1 accumulation has been shown to correlate with the development of distant metastases and a worse prognosis in triple-negative BC (Costa et al., 2018).

A notable finding was the identification of a substantial large-scale transcriptome rearrangement in the riskNF group, which exhibited 7,559 genes that were found to be significantly overexpressed relative to the healthNF group (Figure 6). However, this rearrangement does not affect the expression of CXCL12, which is significantly reduced in riskNF. This phenomenon can be explained by two hypotheses. The expression of CXCL12 in fibroblasts is contingent upon signals from tumor cells or the tumor microenvironment, which are absent in riskNF. Alternatively, the observed reduction in the levels of CXCL12, in riskNF is an independent consequence of the BRCA1 mutation or epigenetic changes that are unrelated to the tumor context. A thorough examination of the extant data reveals an inability to differentiate between these options.

The detection of two healthNF samples with the S1 phenotype is noteworthy (Figure 1). Since S1 is defined as ACTA2^high^ and FAP^high^ using the mean ± SD thresholds, its detection in healthy donors can be explained in two ways. Firstly, normal fibroblasts are known to be heterogeneous, and some of them constitutively express myofibroblast markers at levels that exceed the mean plus one standard deviation for the full dataset. Secondly, a transcriptomic pattern, rather than a functional myofibroblast phenotype, was detected. In the absence of a tumor context, an S1-like pattern may not correspond to the immunosuppressive function described for CAF-S1.

### 4.2 Switching between paracrine and autocrine regulation of CXCL12

The existence of two trajectories of fibroblast activation may be consistent with differences in paracrine and autocrine regulation of CXCL12, providing hypotheses for the interpretation of the observed phenomena rather than demonstrating a specific mechanistic switch. TGF-β1, acting via TGFBR2 → ALK5 → SMAD2/3 phosphorylation → SMAD2/3–SMAD4 complex → nuclear transcription, promotes myofibroblast differentiation (myCAF) with an increase in ACTA2 and FAP (Massagué, 2012; Calon et al., 2014). CXCL12 expression in breast cancer stroma has been demonstrated to be associated with STAT3 activation (Wickramasekara et al., 2026). TGF-β activates STAT3 indirectly through a non-canonical pathway (TAK1 → p38 MAPK → STAT3) or through JAK/STAT-activating cytokines in the microenvironment (Tang et al., 2017).

The hypothesis of Kojima et al. (2010) suggests that the CXCL12/CXCR4 → Gi → PI3K/AKT axis maintains the myofibroblast phenotype. The findings of the present study demonstrate a heightened correlation between CXCL12 and FAP and ITGB1, which was observed in instances of elevated CXCR4 expression, as indicated by the data set. Concurrently, the correlation of CXCR4 with TGF-β1 exhibited a decline in strength. This correlation pattern is consistent with a scenario in which a TGF-β signaling may depend on the CXCR4-associated phenotype. The hypothesis posits that experimental testing is necessary to validate it, and this will be achieved by knocking down CXCR4 in primary CAF-S1/S3/S4 cultures.

The shift in the correlation pattern in accordance with the CXCR4 expression level is of particular interest. In the CXCR4^Neg^ fibroblast population, a high positive correlation was observed between CXCL12 and TGFB1, as well as with differentiation markers including ACTA2, FAP, PDGFRB, and ITGB1 (Figure 7). In the CXCR4^Low/Med^ fibroblast population, the correlation between CXCL12 and TGFB1 reached its maximum levels. In the CXCR4^High^ cohort, the correlation between the levels of the chemokine CXCL12 and TGFB1 became negative. TGFB1 showed a positive correlation with CD44, while the positive correlation between CXCL12 and ITGAV, FAP, and ITGB1 was retained. This pattern suggests that CXCL12 regulation becomes relatively independent of TGF-β signaling at high CXCR4 levels.

The findings of the correlation analysis of bulk RNA-seq data are subject to fundamental limitations. Firstly, the study did not permit the determination of whether or not CXCR4 and CXCL12 are co-expressed in the same cell. Secondly, a change in correlation could either reflect activation of the CXCR4-dependent signaling cascade or be the result of another biological process associated with high CXCR4 expression in a specific CAF subset. As demonstrated in this study, an analysis of these limitations in combination reveals that our observations, based on correlations, should be interpreted as generating a hypothesis rather than serving as direct evidence.

This hypothesis is further substantiated by an additional observation obtained during differential expression analysis. A statistically significant increase in the levels of CXCL12 was recorded in the S1 subtype, even in cases where CXCR4 levels were low or medium. In the presence of elevated CXCR4 levels (CXCR4^High^), a concomitant increase in the expression of CXCL12 was observed across all the subtypes S1, S3, and S4. Subtype S2 with CXCR4^High^ was not detected in the dataset. This precludes the ability to draw conclusions about its behavior under conditions of high CXCR4 expression, which should be considered a limitation when interpreting the results.

### 4.3 Biological interpretation of the CXCR4^High^-CAF transcriptome profile

The elevated expression of CXCR4 and co-expression of its ligand CXCL12 (SDF-1α) are closely associated with paracrine regulation. It has been demonstrated that the interferon response regulates this particular axis, thereby stimulating two key processes, firstly tumor cell invasion and secondly the recruitment of myeloid-derived suppressor or regulatory T-cells (Shi et al., 2020; de Oliveira et al., 2013; Cao et al., 2025). The detected enrichment in Interferon Gamma Response, Interferon Alpha Response, and Allograft Rejection gene sets in CXCR4^High^-CAF suggests the activation of the interferon signaling cascade (Figure 9). The induction of immune surveillance genes (MHC I/II, PD-L1, and CXC9/10) is triggered by IFN-γ via JAK1/2 → STAT1 → IRF1, while IFN-α predominantly acts via IFNAR1/2 → STAT1/STAT2 → IRF9 (Zhou, 2009; Ivashkiv et al., 2014). The observed enrichment of the Allograft Rejection array, which encompasses genes involved in immune recognition and antigen presentation, is consistent with data demonstrating that CAF-S4 expresses MHC II molecules and is capable of activating CD4^+^ T cells (Elyada et al., 2019; Costa et al., 2018). The enrichment of TNF-alpha signaling via NF-kB, inflammatory response, and IL-6/JAK/STAT3 signaling reflects the pro-inflammatory secretory profile characteristic of senescent cells, as evidenced by the secretion of IL-6, IL-8, TNF-α via NF-κB and C/EBPβ (Acosta et al., 2008; Coppé et al., 2010).

Consequently, the enrichment of inflammation-related signatures in the CXCR4^High^-CAF group suggests that these fibroblasts are associated with an inflammatory transcriptional program. CXCR4 has been identified as a critical inflammatory factor. Consequently, it is a promising target for the treatment of autoimmune and infectious diseases that are characterized by the overproduction of pro-inflammatory factors. Such factors include IFNα, TNFα, IL-6 and STAT1/3 (Caspar et al., 2022). Thus, a substantial decline in the aforementioned pro-inflammatory chemokines and cytokines (IFNα, TNFα, IL-6, and STAT1/3 phosphorylation) was observed upon the inhibition of the CXCL12/CXCR4 axis using the CXCR4 antagonist IT1t. This intervention has shown anti-inflammatory properties in a lupus-like model, achieved by modulating IFNα secretion (Smith et al., 2019). Furthermore, a decrease in pro-inflammatory cytokines, such as TNFα and IL-6, was observed in response to Clobenpropit, a small molecule that activates a CXCR4-dependent anti-inflammatory effect (Bekaddour et al., 2021). This effect of CXCR4 inhibitors corroborates the correlation previously identified between the level of CXCR4 and Interferon Response, IL-6/JAK/STAT3 Signaling, and TNF-α Signaling.

*In vitro* studies have demonstrated that the pro-inflammatory cytokine TNF-α, via NF-κB signaling, can enhance CXCR4 expression (Zhi et al., 2015). The observed correlation between CXCR4^High^-CAF and TNF-α/NF-κB expression suggests the possibility of a similar mechanism regulating CXCR4 expression in CAFs. The p53 pathway and Apoptosis sets are enriched in conjunction with the activation of p53-dependent cell cycle arrest and apoptosis programs that accompany cellular senescence. The observed enrichment of KRAS Signaling Up set is compatible with transcriptional patterns reported in oncogene-induced senescence via the RAS → RAF → MEK → ERK pathway. Decreased enrichment of the G2-M Checkpoint, E2F Targets, Myc Targets V1/V2, and Mitotic Spindle sets indicates suppression of proliferative programs in CXCR4^High^-CAF. The diminished enrichment of Epithelial Mesenchymal Transition and TGF-beta Signaling sets is consistent with the altered CXCL12-TGFB1 correlation pattern. Collectively, these patterns delineate a transcriptome profile indicative of a senescent-inflammatory state in CXCR4^High^-CAF. However, these data do not allow us to determine whether this state is a cause or a consequence of high CXCR4 expression. It is also necessary to distinguish between two types of functions of the CXCL12/CXCR4 axis: paracrine (CXCL12 from CAF → CXCR4 of tumor cells → chemotaxis, invasion) (Orimo et al., 2005; Eck et al., 2009; Leonteva et al., 2026) and potentially autocrine (CXCL12 → CXCR4 of the same CAF → maintenance of the myofibroblast phenotype) (Kojima et al., 2010). The data obtained during the course of the study pertain to expression in CAF and do not permit conclusions to be drawn about paracrine effects on tumor cells. In a similar manner, with regard to the putative autocrine function, the bulk RNA-seq data under consideration support only an associative link between high CXCR4 expression and specific co-expression patterns. This finding does not provide direct mechanistic evidence for an autocrine loop.

Crozier et al. (2024) conducted studies that utilized single-cell RNA-seq and spatial transcriptomics, thereby unveiling an additional level of heterogeneity within CAF-S1. This heterogeneity manifested as the presence of multiple subclusters, each exhibiting either an iCAF or a myCAF phenotype. The findings emphasize the complexity of the CAF compartment. The precise quantity of subclusters and their functional characterization are contingent upon the clustering method and the resolution of the analysis. The data presented herein underscore the necessity for further stratification of CAF subtypes in the context of studying the autocrine regulation of CXCR4/CXCL12. Bulk RNA-seq methodologies inherently average the signal across the entirety of the CAF population, thereby precluding the resolution of subcluster heterogeneity. This limitation is further compounded by the fact that the classification of CAF into subtypes S1-S4, which was adapted from a previously published scheme, is based on data for a limited set of markers. While this stratification is advantageous as an analytical instrument, it remains model-based and does not supplant experimental validation at the protein and functional levels. Consequently, any conclusions concerning subtype switching and autocrine secretion of SDF-1α should be regarded as interpretations of the transcriptomic pattern rather than as definitively proven mechanisms.

The present study finds that the inability to unambiguously verify an autocrine loop based on transcriptomic data is consistent with the current state of the literature. A systematic review of the existing literature revealed a paucity of studies directly comparing paracrine and autocrine patterns of CXCL12/CXCR4 activation in the context of CAF differentiation into S1-S4 subtypes.

### 4.4 Study limitations

It is imperative to acknowledge the limitations of this study when interpreting the results.

First, a fundamental limitation is related to the circularity of the classification: the same marker genes (ACTA2, FAP, ITGB1, PDGFRB) are used both to assign samples to subtypes S1-S4 and to test hypotheses about differential expression between these subtypes. According to the established criteria, samples designated as S1 exhibit elevated levels of ACTA2 and FAP, while S4 samples demonstrate high ACTA2 levels and low FAP levels. Consequently, the detection of augmented ACTA2 expression in S1 or diminished FAP in S4 constitutes a rudimentary consequence of the classification process, as opposed to an autonomous biological outcome. It is noteworthy that the genes excluded from the classification rules (namely, CXCL12, CXCR4, TGFB1, CAV1, S100A4, ITGAV, ITGB3, and CD44), along with the correlation patterns and GSEA results, offer independent information. The reliability of the S1-S4 classification in the context of bulk RNA-seq is limited by its reliance on a small set of markers and a specific set of threshold values. The process of Subtype assignment is susceptible to the presence of outliers, variations in dynamic range across studies, and the existence of mixed cell populations within a single sample. Consequently, a proportion of samples may be classified as CAF-S1 or CAF-S4 based on their transcriptomic pattern, without necessarily possessing the full functional repertoire of myofibroblastic or perivascular CAFs *in vivo*. In order to confirm the existence and prevalence of these subtypes in the cohorts under analysis, independent validation is required at the protein level (e.g., multiplex IHC or flow cytometry) and at the single-cell level.

Second, the study is based on a secondary analysis of publicly available transcriptomic data and a systematic literature review. Consequently, its conclusions are associative rather than causal. Statistically significant associations between the chemokine CXCL12, CXCR4, and CAF-phenotype markers do not provide unequivocal evidence that the SDF-1α/CXCR4 autocrine axis is the primary driver of the observed changes, rather than being a component of a broader signaling context.

Third, the transcriptomic analysis integrates datasets from multiple independent studies, which may introduce technical variability (batch effects) arising from differences in library preparation protocols, sequencing platforms, sequencing depth, and primary data processing methods. In the present analysis, all included datasets were processed using a unified analytical pipeline and normalization; however, formal batch effect correction (e.g., ComBat or Harmony) was not applied. Consequently, the influence of technical factors on the observed patterns cannot be entirely ruled out. Nevertheless, the key associations that were identified in this study were found to occur across multiple independent datasets. This finding suggests that the observed batch-effect artefacts are robust, though this does not preclude the possibility of their occurrence. Future validation using prospectively collected and jointly processed samples is desirable.

Fourth, bulk RNA-seq is unable to discern whether the genes CXCR4 and CXCL12 are expressed in the same cell, in different fibroblast subpopulations, or in a mixed population. The detection of co-expression should be interpreted as a characteristic of the population state, not as direct evidence of an intracellular autocrine loop. The existence of strong correlations between markers such as CXCR4, TGFB1, ITGB1, FAP, and others may be indicative of co-activation within the stromal state, rather than direct regulation.

Finally, the Mean ± SD expression level categorization is susceptible to the presence of outliers and does not take into account absolute expression levels. Consequently, this may result in a sample being classified as “High” for a gene with a low absolute level but a narrow range of variation.

When evaluated in its entirety, these limitations suggest that this study should be regarded as a hypothesis-generating exercise, with the aim of elucidating biological scenarios. Rather than providing definitive evidence for the autocrine role of CXCR4/CXCL12 in mammary CAFs, the study aims to facilitate the development of testable hypotheses.

### 4.5 Perspectives

The results of this study formulate specific hypotheses for experimental testing. Initially, it is imperative to verify the autocrine loop of CXCL12/CXCR4 in the primary cultures of CAF-S1, S3, and S4. The hypothesis posits that the knockdown/blockade of CXCR4 by siRNAs or the antagonist (AMD3100) should result in a reduction of ACTA2 and FAP expression, presupposing that the loop is functionally active. Second, it is necessary to establish whether CXCR4 and CXCL12 are expressed in the same cell. This requires scRNA-seq or RNA-FISH on primary CAFs from breast cancer biopsies. Third, the observed reduction of CXCL12 in riskNF with large-scale transcriptomic rearrangement necessitates the testing of the hypothesis that the epigenetic repression of the CXCL12 promoter is caused by a BRCA1 mutation (ChIP-seq for H3K27me3 or DNA methylation). The accumulation of quantitative data on the dynamics of CXCL12, CXCR4 and TGFB1 in primary CAFs under different stimulation conditions will allow for the creation of kinetic models based on a system of ordinary differential equations. These models will assess the conditions under which the autocrine loop becomes self-sustaining and identify key targets (CXCR4, TGFBR1/ALK5, SMAD3) for combined pharmacological action.

Despite the significant role of TGF-β signaling pathways and the CXCL12/CXCR4 axis in fibrosis formation, modern therapy is focused mainly on blocking the TGF-β signaling pathway. This shift is reflected in the abandonment of ineffective monotherapies using small molecule inhibitors (e.g., Galunisertib) and monoclonal antibodies (e.g., Fresolimumab) in favor of advanced clinical trials. These include bispecific antibodies directed at TGF-β/PD-L1 and ligand traps based on soluble TGF-β receptors (sRII or sRIII), and immunoglobulin-fused receptors (TβRII:Fc) (Teixeira et al., 2020; Tapia-Galisteo et al., 2024). Further research will allow us to evaluate the possibility of stratifying therapy by assessing CXCR4 expression as a possible biomarker and developing a direction for inhibiting the CXCL12/CXCR4 axis, which is important for the autocrine regulation of CAF.

## 5 Conclusion

The present study sought to ascertain whether elevated CXCR4 expression in mammary CAFs is associated with altered gene co-expression patterns that are consistent with a shift from paracrine to autocrine regulation of SDF-1α.

High CXCR4 expression was found to be exclusively associated with the CAF group (22.7%, FDR = 5.98 × 10^−4^) and, within CAF, with the S1, S3, and S4 phenotypes, but not S2. The cross-sectional design of the study precludes the determination of whether high CXCR4 expression is a cause or a consequence of terminal differentiation of CAF.

At CXCR4-^High^ levels, a change in the correlation pattern is observed: the CXCL12-TGFB1correlation becomes negative, while CXCL12 maintains a positive correlation with FAP, ITGB1, and ITGAV. This pattern is consistent with a hypothesis that the regulation of CXCL12 in CAF-S1/S3/S4 may shift from TGF-β association to being more strongly linked to CXCR4. However, it does not provide definitive evidence to support this hypothesis, as bulk RNA-seq does not allow for the determination of whether CXCL12 and CXCR4 are expressed in the same cell.

CAFs with CXCR4^high^ levels have been found to be associated with the following gene sets: Interferon gamma response, Interferon alpha response, TNF-alpha signaling via NF-kB, Inflammatory response, IL-6/JAK/STAT3 signaling, Allograft rejection, the p53 pathway, KRAS signaling up, and Apoptosis (GSEA, FDR <0.25). Gene set enrichment may indicate a functional association; however, it does not serve as irrefutable evidence of activation of the corresponding signaling cascades.

A systematic review of the extant literature (34 publications, 2005–2026) revealed that direct experimental evidence for a CXCL12/CXCR4 autocrine loop in CAFs was presented in only one study. The majority of publications did not distinguish between paracrine and autocrine signaling. When considered as a whole, the obtained data formulate a testable hypothesis: in cases of high CXCR4 expression in CAF-S1/S3/S4, the regulation of CXCL12 can shift from a TGF-β-dependent to a CXCR4-mediated autocrine mechanism. Verification of this hypothesis requires CXCR4 knockdown experiments in primary CAFs and scRNA-seq to confirm the co-expression of CXCL12 and CXCR4 at the single-cell level.

## Data Availability

The datasets presented in this study can be found in online repositories. The names of the repository/repositories and accession number(s) can be found in the article/Supplementary Material.
