# Supplementary material for: The CXCL12/CXCR4 axis in breast cancer-associated fibroblasts: a transcriptome analysis and systematic literature review
Source: Front Bioeng Biotechnol. 2026 Oct 12;14:1920541. doi: 10.3389/fbioe.2026.1920541 (PMC13696515; doi:10.3389/fbioe.2026.1920541)
Supplement: Supplementary file 1 [file Supplementaryfile1.docx]

Data Sheet

SRR10263112: healthNF -> Unknown Sample ['Mammary Fibroblast', '', '', '', '', '', ''] -> GSE138734 <https://www.ncbi.nlm.nih.gov/pubmed/34140680>

SRR10263113: healthNF -> Unknown Sample ['Mammary Fibroblast', '', '', '', '', '', ''] -> GSE138734 <https://www.ncbi.nlm.nih.gov/pubmed/34140680>

SRR10263114: healthNF -> Unknown Sample ['Mammary Fibroblast', '', '', '', '', '', ''] -> GSE138734 <https://www.ncbi.nlm.nih.gov/pubmed/34140680>

SRR10263164: healthNF -> Unknown Sample ['Mammary Fibroblast', '', '', '', '', '', ''] -> GSE138734 <https://www.ncbi.nlm.nih.gov/pubmed/34140680>

SRR10263165: healthNF -> Unknown Sample ['Mammary Fibroblast', '', '', '', '', '', ''] -> GSE138734 <https://www.ncbi.nlm.nih.gov/pubmed/34140680>

SRR10263166: healthNF -> Unknown Sample ['Mammary Fibroblast', '', '', '', '', '', ''] -> GSE138734 <https://www.ncbi.nlm.nih.gov/pubmed/34140680>

SRR10263167: healthNF -> Unknown Sample ['Mammary Fibroblast', '', '', '', '', '', ''] -> GSE138734 <https://www.ncbi.nlm.nih.gov/pubmed/34140680>

SRR10263168: healthNF -> GSM4117920: RNAAtlas242_total_RNA; Homo sapiens; RNA-Seq ['Mammary Fibroblast', '', '', '', '', '', ''] -> GSE138734 <https://www.ncbi.nlm.nih.gov/pubmed/34140680>

SRR22086105: healthNF -> GSM6696638: HBF_C01; Homo sapiens; RNA-Seq ['Breast stroma', 'Breast stroma', 'Normal human primary stromal cell line', 'Stromal', '', 'wild type', 'CTRL'] -> GSE216842 No PMID

SRR22086103: healthNF -> GSM6696640: HBF_C03; Homo sapiens; RNA-Seq ['Breast stroma', 'Breast stroma', 'Normal human primary stromal cell line', 'Stromal', '', 'wild type', 'CTRL'] -> GSE216842 No PMID

SRR22086104: healthNF -> GSM6696639: HBF_C02; Homo sapiens; RNA-Seq ['Breast stroma', 'Breast stroma', 'Normal human primary stromal cell line', 'Stromal', '', 'wild type', 'CTRL'] -> GSE216842 No PMID

SRR24019182: CAF -> GSM7124453: 30413-DCIS fibroblast; Homo sapiens; RNA-Seq ['breast ductal carcinoma in situ', 'breast ductal carcinoma in situ', 'primary', 'fibroblast', 'DCIS fibroblast', '', ''] -> GSE228582<https://www.ncbi.nlm.nih.gov/pubmed/37091166>

SRR24019180: CAF -> GSM7124455: 21714-DCIS fibroblast; Homo sapiens; RNA-Seq ['breast ductal carcinoma in situ', 'breast ductal carcinoma in situ', 'primary', 'fibroblast', 'DCIS fibroblast', '', ''] -> GSE228582<https://www.ncbi.nlm.nih.gov/pubmed/37091166>

SRR24019174: CAF -> GSM7124461: 8727- IDC fibroblast; Homo sapiens; RNA-Seq ['invasive breast ductal carcinoma', 'invasive breast ductal carcinoma', 'primary', 'fibroblast', 'IDC fibroblast', '', ''] -> GSE228582<https://www.ncbi.nlm.nih.gov/pubmed/37091166>

SRR24019175: CAF -> GSM7124460: 9019- IDC fibroblast; Homo sapiens; RNA-Seq ['invasive breast ductal carcinoma', 'invasive breast ductal carcinoma', 'primary', 'fibroblast', 'IDC fibroblast', '', ''] -> GSE228582<https://www.ncbi.nlm.nih.gov/pubmed/37091166>

SRR24019184: CAF -> GSM7124451: 80H- DCIS fibroblast; Homo sapiens; RNA-Seq ['breast ductal carcinoma in situ', 'breast ductal carcinoma in situ', 'primary', 'fibroblast', 'DCIS fibroblast', '', ''] -> GSE228582<https://www.ncbi.nlm.nih.gov/pubmed/37091166>

SRR24019177: CAF -> GSM7124458: 2300- IDC fibroblast; Homo sapiens; RNA-Seq ['invasive breast ductal carcinoma', 'invasive breast ductal carcinoma', 'primary', 'fibroblast', 'IDC fibroblast', '', ''] -> GSE228582<https://www.ncbi.nlm.nih.gov/pubmed/37091166>

SRR24019181: CAF -> GSM7124454: 70213-DCIS fibroblast; Homo sapiens; RNA-Seq ['breast ductal carcinoma in situ', 'breast ductal carcinoma in situ', 'primary', 'fibroblast', 'DCIS fibroblast', '', ''] -> GSE228582<https://www.ncbi.nlm.nih.gov/pubmed/37091166>

SRR24019171: healthNF -> GSM7124464: 2525-normal breast fibroblast; Homo sapiens; RNA-Seq ['normal breast', 'normal breast', 'primary', 'fibroblast', 'normal breast fibroblast', '', ''] -> GSE228582<https://www.ncbi.nlm.nih.gov/pubmed/37091166>

SRR24019176: CAF -> GSM7124459: 2760- IDC fibroblast; Homo sapiens; RNA-Seq ['invasive breast ductal carcinoma', 'invasive breast ductal carcinoma', 'primary', 'fibroblast', 'IDC fibroblast', '', ''] -> GSE228582<https://www.ncbi.nlm.nih.gov/pubmed/37091166>

SRR24019178: CAF -> GSM7124457: 8661- IDC fibroblast; Homo sapiens; RNA-Seq ['invasive breast ductal carcinoma', 'invasive breast ductal carcinoma', 'primary', 'fibroblast', 'IDC fibroblast', '', ''] -> GSE228582<https://www.ncbi.nlm.nih.gov/pubmed/37091166>

SRR24019179: CAF -> GSM7124456: 8870- IDC fibroblast; Homo sapiens; RNA-Seq ['invasive breast ductal carcinoma', 'invasive breast ductal carcinoma', 'primary', 'fibroblast', 'IDC fibroblast', '', ''] -> GSE228582<https://www.ncbi.nlm.nih.gov/pubmed/37091166>

SRR24019173: cancerNF -> GSM7124462: 9019- normal adjacent breast fibroblast; Homo sapiens; RNA-Seq ['normal adjacent breast to IDC', 'normal adjacent breast to IDC', 'primary', 'fibroblast', 'normal adjacent breast fibroblast', '', ''] -> GSE228582 <https://www.ncbi.nlm.nih.gov/pubmed/37091166>

SRR24019183: CAF -> GSM7124452: 1213249-DCIS fibroblast; Homo sapiens; RNA-Seq ['breast ductal carcinoma in situ', 'breast ductal carcinoma in situ', 'primary', 'fibroblast', 'DCIS fibroblast', '', ''] -> GSE228582<https://www.ncbi.nlm.nih.gov/pubmed/37091166>

SRR24019170: healthNF -> GSM7124465: 4009- normal breast fibroblast; Homo sapiens; RNA-Seq ['normal breast', 'normal breast', 'primary', 'fibroblast', 'normal breast fibroblast', '', ''] -> GSE228582<https://www.ncbi.nlm.nih.gov/pubmed/37091166>

SRR24019185: CAF -> GSM7124450: 102115- DCIS fibroblast; Homo sapiens; RNA-Seq ['breast ductal carcinoma in situ', 'breast ductal carcinoma in situ', 'primary', 'fibroblast', 'DCIS fibroblast', '', ''] -> GSE228582<https://www.ncbi.nlm.nih.gov/pubmed/37091166>

SRR24019172: cancerNF -> GSM7124463: 8727-normal adjacent breast fibroblast; Homo sapiens; RNA-Seq ['normal adjacent breast to IDC', 'normal adjacent breast to IDC', 'primary', 'fibroblast', 'normal adjacent breast fibroblast', '', ''] -> GSE228582 <https://www.ncbi.nlm.nih.gov/pubmed/37091166>

SRR24422180: CAF -> GSM7291339: CAFs siCon4; Homo sapiens; RNA-Seq ['Primary human breast cancer-associated fibroblasts (CAFs)', '', '', 'Primary human breast cancer-associated fibroblasts (CAFs)', '', '', 'si-Control'] -> GSE231594 No PMID

SRR24422183: CAF -> GSM7291336: CAFs siCon1; Homo sapiens; RNA-Seq ['Primary human breast cancer-associated fibroblasts (CAFs)', '', '', 'Primary human breast cancer-associated fibroblasts (CAFs)', '', '', 'si-Control'] -> GSE231594 No PMID

SRR24422181: CAF -> GSM7291338: CAFs siCon3; Homo sapiens; RNA-Seq ['Primary human breast cancer-associated fibroblasts (CAFs)', '', '', 'Primary human breast cancer-associated fibroblasts (CAFs)', '', '', 'si-Control'] -> GSE231594 No PMID

SRR24422182: CAF -> GSM7291337: CAFs siCon2; Homo sapiens; RNA-Seq ['Primary human breast cancer-associated fibroblasts (CAFs)', '', '', 'Primary human breast cancer-associated fibroblasts (CAFs)', '', '', 'si-Control'] -> GSE231594 No PMID

SRR29325432: CAF -> GSM8314942: HMCAF in CCR region, rep2; Homo sapiens; RNA-Seq ['GelMA and gelatin hydrogel', 'GelMA and gelatin hydrogel', 'HMCAF', 'human mammary cancer-associated fibroblasts', '', 'WT', '3D printing in CCR region'] -> GSE269415 <https://www.ncbi.nlm.nih.gov/pubmed/39112274>

SRR29325433: CAF -> GSM8314941: HMCAF in CCR region; Homo sapiens; RNA-Seq ['GelMA and gelatin hydrogel', 'GelMA and gelatin hydrogel', 'HMCAF', 'human mammary cancer-associated fibroblasts', '', 'WT', '3D printing in CCR region'] -> GSE269415 <https://www.ncbi.nlm.nih.gov/pubmed/39112274>

SRR29325429: CAF -> GSM8314945: HMCAF in SR region; Homo sapiens; RNA-Seq ['GelMA and gelatin hydrogel', 'GelMA and gelatin hydrogel', 'HMCAF', 'human mammary cancer-associated fibroblasts', '', 'WT', '3D printing in SR region'] -> GSE269415 <https://www.ncbi.nlm.nih.gov/pubmed/39112274>

SRR29325428: CAF -> GSM8314946: HMCAF in SR region, rep2; Homo sapiens; RNA-Seq ['GelMA and gelatin hydrogel', 'GelMA and gelatin hydrogel', 'HMCAF', 'human mammary cancer-associated fibroblasts', '', 'WT', '3D printing in SR region'] -> GSE269415 <https://www.ncbi.nlm.nih.gov/pubmed/39112274>

SRR29428548: CAF -> GSM8331617: 1370820; Homo sapiens; RNA-Seq ['Breast cancer-associated fibroblast', '', '', 'Breast cancer-associated fibroblast', '', '', ''] -> GSE269968 <https://www.ncbi.nlm.nih.gov/pubmed/38937754>

SRR29428547: CAF -> GSM8331618: 1370821; Homo sapiens; RNA-Seq ['Breast cancer-associated fibroblast', '', '', 'Breast cancer-associated fibroblast', '', '', ''] -> GSE269968 <https://www.ncbi.nlm.nih.gov/pubmed/38937754>

SRR29428549: CAF -> GSM8331616: 1370819; Homo sapiens; RNA-Seq ['Breast cancer-associated fibroblast', '', '', 'Breast cancer-associated fibroblast', '', '', ''] -> GSE269968 <https://www.ncbi.nlm.nih.gov/pubmed/38937754>

SRR33430782: cancerNF -> GSM8968950: Patient 4, NF, 4117; Homo sapiens; RNA-Seq ['Breast', 'Breast', '', 'Fibroblasts', '', 'WT', ''] -> GSE296349 <https://www.ncbi.nlm.nih.gov/pubmed/40598415>

SRR33430784: cancerNF -> GSM8968948: Patient 3, NF, 4113; Homo sapiens; RNA-Seq ['Breast', 'Breast', '', 'Fibroblasts', '', 'WT', ''] -> GSE296349 <https://www.ncbi.nlm.nih.gov/pubmed/40598415>

SRR33430777: CAF -> GSM8968955: Patient 7, CAF, 4340; Homo sapiens; RNA-Seq ['Breast', 'Breast', '', 'Fibroblasts', '', 'WT', ''] -> GSE296349 <https://www.ncbi.nlm.nih.gov/pubmed/40598415>

SRR33430785: CAF -> GSM8968947: Patient 3, CAF, 4112; Homo sapiens; RNA-Seq ['Breast', 'Breast', '', 'Fibroblasts', '', 'WT', ''] -> GSE296349 <https://www.ncbi.nlm.nih.gov/pubmed/40598415>

SRR33430778: cancerNF -> GSM8968954: Patient 6, NF, 4316; Homo sapiens; RNA-Seq ['Breast', 'Breast', '', 'Fibroblasts', '', 'WT', ''] -> GSE296349 <https://www.ncbi.nlm.nih.gov/pubmed/40598415>

SRR33430779: CAF -> GSM8968953: Patient 6, CAF, 4315; Homo sapiens; RNA-Seq ['Breast', 'Breast', '', 'Fibroblasts', '', 'WT', ''] -> GSE296349 <https://www.ncbi.nlm.nih.gov/pubmed/40598415>

SRR33430770: cancerNF -> GSM8968962: Patient 10, NFF, 3537; Homo sapiens; RNA-Seq ['Breast', 'Breast', '', 'Fibroblasts', '', 'WT', ''] -> GSE296349 <https://www.ncbi.nlm.nih.gov/pubmed/40598415>

SRR33430767: CAF -> GSM8968965: Patient 12, CAF, 4722; Homo sapiens; RNA-Seq ['Breast', 'Breast', '', 'Fibroblasts', '', 'WT', ''] -> GSE296349 <https://www.ncbi.nlm.nih.gov/pubmed/40598415>

SRR33430786: cancerNF -> GSM8968946: Patient 2, NF, 4028; Homo sapiens; RNA-Seq ['Breast', 'Breast', '', 'Fibroblasts', '', 'WT', ''] -> GSE296349 <https://www.ncbi.nlm.nih.gov/pubmed/40598415>

SRR33430783: CAF -> GSM8968949: Patient 4, CAF, 4116; Homo sapiens; RNA-Seq ['Breast', 'Breast', '', 'Fibroblasts', '', 'WT', ''] -> GSE296349 <https://www.ncbi.nlm.nih.gov/pubmed/40598415>

SRR33430788: cancerNF -> GSM8968944: Patient 1, NF, 4034; Homo sapiens; RNA-Seq ['Breast', 'Breast', '', 'Fibroblasts', '', 'WT', ''] -> GSE296349 <https://www.ncbi.nlm.nih.gov/pubmed/40598415>

SRR33430769: CAF -> GSM8968963: Patient 11, CAF, 4299; Homo sapiens; RNA-Seq ['Breast', 'Breast', '', 'Fibroblasts', '', 'WT', ''] -> GSE296349 <https://www.ncbi.nlm.nih.gov/pubmed/40598415>

SRR33430774: cancerNF -> GSM8968958: Patient 8, NF, 4344; Homo sapiens; RNA-Seq ['Breast', 'Breast', '', 'Fibroblasts', '', 'WT', ''] -> GSE296349 <https://www.ncbi.nlm.nih.gov/pubmed/40598415>

SRR33430768: cancerNF -> GSM8968964: Patient 11, NF, 4300; Homo sapiens; RNA-Seq ['Breast', 'Breast', '', 'Fibroblasts', '', 'WT', ''] -> GSE296349 <https://www.ncbi.nlm.nih.gov/pubmed/40598415>

SRR33430787: CAF -> GSM8968945: Patient 2, CAF, 4027; Homo sapiens; RNA-Seq ['Breast', 'Breast', '', 'Fibroblasts', '', 'WT', ''] -> GSE296349 <https://www.ncbi.nlm.nih.gov/pubmed/40598415>

SRR33430781: CAF -> GSM8968951: Patient 5, CAF, 4214; Homo sapiens; RNA-Seq ['Breast', 'Breast', '', 'Fibroblasts', '', 'WT', ''] -> GSE296349 <https://www.ncbi.nlm.nih.gov/pubmed/40598415>

SRR33430775: CAF -> GSM8968957: Patient 8, CAF, 4344; Homo sapiens; RNA-Seq ['Breast', 'Breast', '', 'Fibroblasts', '', 'WT', ''] -> GSE296349 <https://www.ncbi.nlm.nih.gov/pubmed/40598415>

SRR33430771: CAF -> GSM8968961: Patient 10, CAF, 3536; Homo sapiens; RNA-Seq ['Breast', 'Breast', '', 'Fibroblasts', '', 'WT', ''] -> GSE296349 <https://www.ncbi.nlm.nih.gov/pubmed/40598415>

SRR33430780: cancerNF -> GSM8968952: Patient 5, NF, 4215; Homo sapiens; RNA-Seq ['Breast', 'Breast', '', 'Fibroblasts', '', 'WT', ''] -> GSE296349 <https://www.ncbi.nlm.nih.gov/pubmed/40598415>

SRR33430772: cancerNF -> GSM8968960: Patient 9, NF, 3533; Homo sapiens; RNA-Seq ['Breast', 'Breast', '', 'Fibroblasts', '', 'WT', ''] -> GSE296349 <https://www.ncbi.nlm.nih.gov/pubmed/40598415>

SRR33430766: cancerNF -> GSM8968966: Patient 12, NF, 4723; Homo sapiens; RNA-Seq ['Breast', 'Breast', '', 'Fibroblasts', '', 'WT', ''] -> GSE296349 <https://www.ncbi.nlm.nih.gov/pubmed/40598415>

SRR33430789: CAF -> GSM8968943: Patient 1, CAF, 4033; Homo sapiens; RNA-Seq ['Breast', 'Breast', '', 'Fibroblasts', '', 'WT', ''] -> GSE296349 <https://www.ncbi.nlm.nih.gov/pubmed/40598415>

SRR33430773: CAF -> GSM8968959: Patient 9, CAF, 3532; Homo sapiens; RNA-Seq ['Breast', 'Breast', '', 'Fibroblasts', '', 'WT', ''] -> GSE296349 <https://www.ncbi.nlm.nih.gov/pubmed/40598415>

SRR33430776: cancerNF -> GSM8968956: Patient 7, NF, 4341; Homo sapiens; RNA-Seq ['Breast', 'Breast', '', 'Fibroblasts', '', 'WT', ''] -> GSE296349 <https://www.ncbi.nlm.nih.gov/pubmed/40598415>

SRR33548902: riskNF -> GSM8980856: 48539_051L_pos; Homo sapiens; RNA-Seq ['human peri-epithelial breast fibroblast', '', '', 'human peri-epithelial breast fibroblast', '', '', ''] -> GSE296967<https://www.ncbi.nlm.nih.gov/pubmed/40375322>

SRR33548884: riskNF -> GSM8980874: 48557_122_pos; Homo sapiens; RNA-Seq ['human peri-epithelial breast fibroblast', '', '', 'human peri-epithelial breast fibroblast', '', '', ''] -> GSE296967<https://www.ncbi.nlm.nih.gov/pubmed/40375322>

SRR33548890: riskNF -> GSM8980868: 48551_117_pos; Homo sapiens; RNA-Seq ['human peri-epithelial breast fibroblast', '', '', 'human peri-epithelial breast fibroblast', '', '', ''] -> GSE296967<https://www.ncbi.nlm.nih.gov/pubmed/40375322>

SRR33548908: riskNF -> GSM8980850: 48533_240_pos; Homo sapiens; RNA-Seq ['human peri-epithelial breast fibroblast', '', '', 'human peri-epithelial breast fibroblast', '', '', ''] -> GSE296967<https://www.ncbi.nlm.nih.gov/pubmed/40375322>

SRR33548907: riskNF -> GSM8980851: 48534_C078-; Homo sapiens; RNA-Seq ['human peri-epithelial breast fibroblast', '', '', 'human peri-epithelial breast fibroblast', '', '', ''] -> GSE296967<https://www.ncbi.nlm.nih.gov/pubmed/40375322>

SRR33548906: riskNF -> GSM8980852: 48535_C078_pos; Homo sapiens; RNA-Seq ['human peri-epithelial breast fibroblast', '', '', 'human peri-epithelial breast fibroblast', '', '', ''] -> GSE296967<https://www.ncbi.nlm.nih.gov/pubmed/40375322>

SRR33548882: riskNF -> GSM8980876: 48559_C045_pos; Homo sapiens; RNA-Seq ['human peri-epithelial breast fibroblast', '', '', 'human peri-epithelial breast fibroblast', '', '', ''] -> GSE296967<https://www.ncbi.nlm.nih.gov/pubmed/40375322>

SRR33548883: riskNF -> GSM8980875: 48558_C045-; Homo sapiens; RNA-Seq ['human peri-epithelial breast fibroblast', '', '', 'human peri-epithelial breast fibroblast', '', '', ''] -> GSE296967<https://www.ncbi.nlm.nih.gov/pubmed/40375322>

SRR33548893: riskNF -> GSM8980865: 48548_169-; Homo sapiens; RNA-Seq ['human peri-epithelial breast fibroblast', '', '', 'human peri-epithelial breast fibroblast', '', '', ''] -> GSE296967 <https://www.ncbi.nlm.nih.gov/pubmed/40375322>

SRR33548909: riskNF -> GSM8980849: 48532_240-; Homo sapiens; RNA-Seq ['human peri-epithelial breast fibroblast', '', '', 'human peri-epithelial breast fibroblast', '', '', ''] -> GSE296967 <https://www.ncbi.nlm.nih.gov/pubmed/40375322>

SRR33548900: riskNF -> GSM8980858: 48541_C073_pos; Homo sapiens; RNA-Seq ['human peri-epithelial breast fibroblast', '', '', 'human peri-epithelial breast fibroblast', '', '', ''] -> GSE296967<https://www.ncbi.nlm.nih.gov/pubmed/40375322>

SRR33548887: riskNF -> GSM8980871: 48554_C094-; Homo sapiens; RNA-Seq ['human peri-epithelial breast fibroblast', '', '', 'human peri-epithelial breast fibroblast', '', '', ''] -> GSE296967<https://www.ncbi.nlm.nih.gov/pubmed/40375322>

SRR33548910: riskNF -> GSM8980848: 48531_160_pos; Homo sapiens; RNA-Seq ['human peri-epithelial breast fibroblast', '', '', 'human peri-epithelial breast fibroblast', '', '', ''] -> GSE296967<https://www.ncbi.nlm.nih.gov/pubmed/40375322>

SRR33548905: riskNF -> GSM8980853: 48536_C145-; Homo sapiens; RNA-Seq ['human peri-epithelial breast fibroblast', '', '', 'human peri-epithelial breast fibroblast', '', '', ''] -> GSE296967<https://www.ncbi.nlm.nih.gov/pubmed/40375322>

SRR33548898: riskNF -> GSM8980860: 48543_C051_pos; Homo sapiens; RNA-Seq ['human peri-epithelial breast fibroblast', '', '', 'human peri-epithelial breast fibroblast', '', '', ''] -> GSE296967<https://www.ncbi.nlm.nih.gov/pubmed/40375322>

SRR33548901: riskNF -> GSM8980857: 48540_C073-; Homo sapiens; RNA-Seq ['human peri-epithelial breast fibroblast', '', '', 'human peri-epithelial breast fibroblast', '', '', ''] -> GSE296967<https://www.ncbi.nlm.nih.gov/pubmed/40375322>

SRR33548892: riskNF -> GSM8980866: 48549_169_pos; Homo sapiens; RNA-Seq ['human peri-epithelial breast fibroblast', '', '', 'human peri-epithelial breast fibroblast', '', '', ''] -> GSE296967<https://www.ncbi.nlm.nih.gov/pubmed/40375322>

SRR33548904: riskNF -> GSM8980854: 48537_C145_pos; Homo sapiens; RNA-Seq ['human peri-epithelial breast fibroblast', '', '', 'human peri-epithelial breast fibroblast', '', '', ''] -> GSE296967<https://www.ncbi.nlm.nih.gov/pubmed/40375322>

SRR33548903: riskNF -> GSM8980855: 48538_051L-; Homo sapiens; RNA-Seq ['human peri-epithelial breast fibroblast', '', '', 'human peri-epithelial breast fibroblast', '', '', ''] -> GSE296967<https://www.ncbi.nlm.nih.gov/pubmed/40375322>

SRR33548911: riskNF -> GSM8980847: 48530_160-; Homo sapiens; RNA-Seq ['human peri-epithelial breast fibroblast', '', '', 'human peri-epithelial breast fibroblast', '', '', ''] -> GSE296967 <https://www.ncbi.nlm.nih.gov/pubmed/40375322>

SRR33548889: riskNF -> GSM8980869: 48552_112-; Homo sapiens; RNA-Seq ['human peri-epithelial breast fibroblast', '', '', 'human peri-epithelial breast fibroblast', '', '', ''] -> GSE296967 <https://www.ncbi.nlm.nih.gov/pubmed/40375322>

SRR33548894: riskNF -> GSM8980864: 48547_C023_pos; Homo sapiens; RNA-Seq ['human peri-epithelial breast fibroblast', '', '', 'human peri-epithelial breast fibroblast', '', '', ''] -> GSE296967<https://www.ncbi.nlm.nih.gov/pubmed/40375322>

SRR33548895: riskNF -> GSM8980863: 48546_C023-; Homo sapiens; RNA-Seq ['human peri-epithelial breast fibroblast', '', '', 'human peri-epithelial breast fibroblast', '', '', ''] -> GSE296967<https://www.ncbi.nlm.nih.gov/pubmed/40375322>

SRR33548888: riskNF -> GSM8980870: 48553_112_pos; Homo sapiens; RNA-Seq ['human peri-epithelial breast fibroblast', '', '', 'human peri-epithelial breast fibroblast', '', '', ''] -> GSE296967<https://www.ncbi.nlm.nih.gov/pubmed/40375322>

SRR33548885: riskNF -> GSM8980873: 48556_122-; Homo sapiens; RNA-Seq ['human peri-epithelial breast fibroblast', '', '', 'human peri-epithelial breast fibroblast', '', '', ''] -> GSE296967 <https://www.ncbi.nlm.nih.gov/pubmed/40375322>

SRR33548899: riskNF -> GSM8980859: 48542_C051-; Homo sapiens; RNA-Seq ['human peri-epithelial breast fibroblast', '', '', 'human peri-epithelial breast fibroblast', '', '', ''] -> GSE296967<https://www.ncbi.nlm.nih.gov/pubmed/40375322>

SRR33548891: riskNF -> GSM8980867: 48550_117-; Homo sapiens; RNA-Seq ['human peri-epithelial breast fibroblast', '', '', 'human peri-epithelial breast fibroblast', '', '', ''] -> GSE296967 <https://www.ncbi.nlm.nih.gov/pubmed/40375322>

SRR33548886: riskNF -> GSM8980872: 48555_C094_pos; Homo sapiens; RNA-Seq ['human peri-epithelial breast fibroblast', '', '', 'human peri-epithelial breast fibroblast', '', '', ''] -> GSE296967<https://www.ncbi.nlm.nih.gov/pubmed/40375322>

SRR33548896: riskNF -> GSM8980862: 48545_C147_pos; Homo sapiens; RNA-Seq ['human peri-epithelial breast fibroblast', '', '', 'human peri-epithelial breast fibroblast', '', '', ''] -> GSE296967<https://www.ncbi.nlm.nih.gov/pubmed/40375322>

SRR33548897: riskNF -> GSM8980861: 48544_C147-; Homo sapiens; RNA-Seq ['human peri-epithelial breast fibroblast', '', '', 'human peri-epithelial breast fibroblast', '', '', ''] -> GSE296967<https://www.ncbi.nlm.nih.gov/pubmed/40375322>

SRR34230819: CAF -> GSM9072568: Female breast cancer-associated fibroblast, 1362233; Homo sapiens; RNA-Seq ['Female breast cancer-associated fibroblast', '', '', 'Female breast cancer-associated fibroblast', '', '', ''] -> GSE300947 <https://www.ncbi.nlm.nih.gov/pubmed/41088372>

SRR34230820: CAF -> GSM9072567: Female breast cancer-associated fibroblast, 1362232; Homo sapiens; RNA-Seq ['Female breast cancer-associated fibroblast', '', '', 'Female breast cancer-associated fibroblast', '', '', ''] -> GSE300947 <https://www.ncbi.nlm.nih.gov/pubmed/41088372>

SRR34230821: CAF -> GSM9072566: Female breast cancer-associated fibroblast, 1362231; Homo sapiens; RNA-Seq ['Female breast cancer-associated fibroblast', '', '', 'Female breast cancer-associated fibroblast', '', '', ''] -> GSE300947 <https://www.ncbi.nlm.nih.gov/pubmed/41088372>

SRR34789202: CAF -> GSM9143699: Breast CAFs DMSO RNA Replicate 1; Homo sapiens; RNA-Seq ['fibroblast', '', 'fibroblast', 'Cell line', '', 'WT', 'DMSO'] -> GSE304108 <https://www.ncbi.nlm.nih.gov/pubmed/40975064>

SRR34789197: CAF -> GSM9143704: Breast CAFs DDR1/2, MAPK12 Non-targeting Control RNA Replicate 2; Homo sapiens; RNA-Seq ['fibroblast', '', 'fibroblast', 'Cell line', '', 'WT', 'Non-targeting Control'] -> GSE304108<https://www.ncbi.nlm.nih.gov/pubmed/40975064>

SRR34789190: CAF -> GSM9143711: Breast CAFs DMSO Replicate 1; Homo sapiens; RNA-Seq ['fibroblast', '', 'fibroblast', 'Cell line', '', 'WT', 'DMSO'] -> GSE304108 <https://www.ncbi.nlm.nih.gov/pubmed/40975064>

SRR34789198: CAF -> GSM9143703: Breast CAFs DDR1/2, MAPK12 Non-targeting Control RNA Replicate 1; Homo sapiens; RNA-Seq ['fibroblast', '', 'fibroblast', 'Cell line', '', 'WT', 'Non-targeting Control'] -> GSE304108<https://www.ncbi.nlm.nih.gov/pubmed/40975064>

SRR34789189: CAF -> GSM9143712: Breast CAFs DMSO Replicate 2; Homo sapiens; RNA-Seq ['fibroblast', '', 'fibroblast', 'Cell line', '', 'WT', 'DMSO'] -> GSE304108 <https://www.ncbi.nlm.nih.gov/pubmed/40975064>

SRR34789201: CAF -> GSM9143700: Breast CAFs DMSO RNA Replicate 2; Homo sapiens; RNA-Seq ['fibroblast', '', 'fibroblast', 'Cell line', '', 'WT', 'DMSO'] -> GSE304108 <https://www.ncbi.nlm.nih.gov/pubmed/40975064>
