# Supplementary material for: The CXCL12/CXCR4 axis in breast cancer-associated fibroblasts: a transcriptome analysis and systematic literature review
Source: Front Bioeng Biotechnol. 2026 Oct 12;14:1920541. doi: 10.3389/fbioe.2026.1920541 (PMC13696515; doi:10.3389/fbioe.2026.1920541)
Supplement: Supplementary file 2 [file Supplementaryfile2.docx]

Supplementary Material

### Systematic Literature Review Strategy

### 1. Research Question

The research question was formulated using a modified PICO framework adapted for a systematic review with elements of a transcriptomic study. **P** (population): human mammary gland CAF; **I** (intervention/exposure): co-expression of CXCR4 and CXCL12 in CAF; **C** (comparator): paracrine model (CXCL12 secreted by CAF acts on CXCR4 in tumor cells); **O** (outcome): presence/absence of autocrine loop involving CXCR4 and CXCL12 in CAF.

### 2. Literature Search Sources and Period

The literature search was performed using the PubMed and Embase electronic databases from 2005 to 2026. These databases were selected due to their comprehensive coverage of peer-reviewed biomedical literature and availability of structured metadata. The search query was constructed using thematic blocks of keywords connected by the logical operator "AND." Full search query: *("breast cancer*"[Title/Abstract] OR "breast carcinoma*"[Title/Abstract] OR "breast neoplasm*"[Title/Abstract] OR "breast tumor*"[Title/Abstract] OR "mammary cancer*"[Title/Abstract] OR "mammary carcinoma*"[Title/Abstract] OR "mammary neoplasm*"[Title/Abstract]) AND ("cancer-associated fibroblast*"[Title/Abstract] OR "CAF*"[Title/Abstract] OR "stromal fibroblast*"[Title/Abstract]) AND ("CXCR4")*.

Limiting the search to a single database (PubMed) and open SRA/GEO data ensures reproducibility, though it does not eliminate publication bias or incomplete literature coverage.

### 3. Publication Inclusion and Exclusion Criteria

Inclusion criteria: original studies or reviews; subject - CAFs or human mammary stromal fibroblasts; CXCL12/CXCR4 axis; publication in English.

Exclusion criteria: studies on cell lines lacking primary CAFs; studies in which CXCL12/CXCR4 is mentioned solely as a marker without undergoing functional analysis; conference proceedings, abstracts, and preprints that did not undergo peer review.

### 4. Selection Process and Qualitative Synthesis

The selection process was conducted in accordance with a systematic protocol. Initially, the duplicates were eliminated. Subsequently, the titles and abstracts were subjected to a screening process by two independent reviewers. Subsequently, a comprehensive analysis of the full text of potentially relevant articles was conducted. Disagreements were resolved by consensus or by a third reviewer. The results of the systematic review were summarized in tabular form, indicating the DOI and year of publication.

Importantly, studies reporting positive, negative, null, divergent, or inconclusive findings were retained if they met the predefined eligibility criteria. During qualitative synthesis, each study was categorized as providing: direct experimental evidence for an autocrine CXCL12/CXCR4 loop in CAFs; indirect evidence compatible with a possible autocrine mechanism; evidence supporting a paracrine model in which CAF-derived CXCL12 acts on CXCR4-expressing cells in the tumor microenvironment; or no specification of signaling mode. Thus, contrasting findings were evaluated according to their study context and level of evidence rather than treated as grounds for exclusion.

Due to the substantial heterogeneity in design, measured outcomes, and data formats of the included publications, conducting a classical quantitative meta-analysis was not feasible. Instead, a qualitative synthesis (i.e., thematic synthesis) of associations between the status of CXCR4 and the presence of autocrine secretion of SDF-1α (CXCL12) in fibroblasts was performed. To assess the quality of the included studies, an adapted set of criteria was used, taking into account the heterogeneity of design (functional, transcriptomic, and association studies):

- The identification of the CAF/fibroblast cell population must be clear;
- The availability of validated markers and signatures specific to CAFs is necessary;
- The detection method must be reproducible (e.g., PCR, IHC, flow cytometry, RNA-seq);
- Appropriate controls (e.g., normal fibroblasts, negative control) must be available;
- The indication of the signaling type (e.g., autocrine, paracrine, unspecified) is essential.

### Transcriptome Data Analysis Methodology

### 1. Categorization of Expression Levels and Classification of S1-S4 Subtypes

The classification strategy employed was adapted from a framework originally proposed by Costa et al. for breast cancer, and was selected for several reasons. Firstly, the study is based on a compact panel of six well-characterised CAF markers (ACTA2/α-SMA, FAP, ITGB1/CD29, PDGFRB, CAV1, S100A4/FSP1), which are reliably detected in bulk RNA-seq datasets and are traditionally used to distinguish quiescent fibroblasts from various activated phenotypes (myofibroblastic, matrix-remodelling, perivascular). Secondly, the utilisation of a previously published and clinically interpretable classification system facilitates the biological interpretation of the results and comparison with existing studies of CAF heterogeneity at the single-cell and histological levels.

Concurrently, the application of a subtyping scheme originally derived from multiparametric flow cytometry and immunohistochemistry to bulk RNA-seq data is encumbered by fundamental limitations. In this study, the designations S1-S4 should be regarded as a model stratification of transcriptomic profiles according to the relative expression levels of marker genes, rather than as evidence that each sample consists of a homogeneous population of CAFs with corresponding functional properties. Given that the mean expression of RNA-seq is calculated from a composite sample of fibroblasts, the designation of a particular subtype may be indicative of the prevalent transcriptomic pattern within the sample, as opposed to the state of individual cells.

The expression levels of each marker gene were then categorized using the Mean ± SD method into four levels:

- Neg: value ≤ Mean - 1SD
- Low: value in the interval (Mean - 1SD, Mean];
- Med: value in the interval (Mean, Mean + 1SD];
- High: value > Mean + 1SD.

This method is vulnerable to outliers and does not take into account the absolute expression level. However, it ensures reproducibility of stratification and is consistent with the approach used in the original classification by Costa et al. (2018). The classification of samples into S1-S4 subtypes was performed using the adapted algorithm of Costa et al. (2018) based on the categorized levels of six marker genes:

- S1: ACTA2 ∈ {Med, High} & FAP ∈ {Med, High}
- S2: ACTA2 ∈ {Neg, Low} & (ITGB1 ∈ {Neg, Low} | PDGFRB ∈ {Neg, Low})
- S3: ACTA2 ∈ {Neg, Low} & (ITGB1 = Med | PDGFRB = Med)
- S4: ACTA2 ∈ {Med, High} & FAP ∈ {Neg, Low}

### 2. Differential Expression Analysis

The estimation of differential expression (DE) was conducted using the DESeq2 (v1.38.3) approach, which implements a negative binomial distribution model with variance shrinkage estimation (Love et al., 2014). Gene-level aggregated est_counts from Kallisto were imported into DESeq2 using tximport (v1.26.0). For each comparison, the following calculations were made:

- *log2 fold change* (log2FC): the logarithm of the ratio of mean expression levels between groups B and A, with a shrinkage estimate (apeShrinkage) to stabilize values at a small number of counts;
- *p-value*: the probability of the observed difference under the null hypothesis of no change in expression;
- *adjusted p-value* (padj): the p-value corrected for multiple testing using the Benjamini-Hochberg method (FDR). Significance thresholds: The statistical test was performed using the following criteria: a log2FC greater than 1.0, a p-value less than 0.05, and an FDR p-value less than 0.05.

### 3. Principal Component Analysis (PCA)

PCA was performed on the log2(TPM+1) matrix for all filtered genes. The visualization incorporated a color coding system that categorized clinical and morphological groups and S1-S4 subtypes, with dot size proportional to expression level.

### 4. Contingency Tables and Residual Analysis

Contingency tables were constructed to assess the association between clinical and morphological groups and S1-S4 subtypes. The global significance of the study's findings was assessed using the χ² test, with a significance level of p < 0.05. To identify cells with significant deviations from expected frequencies, standardized residuals were calculated (Residual = (Observed - Expected) /√Expected; |Residual| > 2). The Benjamini-Hochberg correction for multiple cell comparisons yielded an FDR of less than 0.05. For expected frequencies less than 5, Fisher's exact test or the Monte Carlo method (B = 10,000) was employed.

### 5. Correlation Analysis

Correlations between marker genes were assessed using the Spearman's rho coefficient. The statistical significance threshold was set at p < 0.05. Correlation matrices were constructed for the total sample and for clinical and morphological groups, as well as for CXCR4 levels.

### 6. Gene Set Enrichment Analysis (GSEA)

In order to facilitate the biological interpretation of transcriptomic differences between fibroblast groups, the GSEA PreRanked algorithm was utilized (Subramanian et al., 2005). The input comprised a comprehensive catalog of genes, meticulously arranged based on their shrunken log2FC values derived from the DESeq2 analysis. For each gene set from the MSigDB Hallmark 2020 collection (Liberzon et al., 2015), the following were calculated:

- *Enrichment Score* (ES): The degree to which a set of genes is overrepresented in the upper or lower parts of a ranked list.
- *Normalized Enrichment Score* (NES): A standardized indicator that allows for the comparison of pathways of different sizes. A positive NES indicates pathway activation in the experimental group, while a negative NES indicates pathway activation in the baseline group.
- *FDR q-value*: A statistical significance that is adjusted for multiple testing. The significance threshold is FDR < 0.25. In the GSEA plot, categories are ordered by the average NES value of their constituent pathways. The x-axis of the graph represents the NES value, with the dot size reflecting the proportion of genes in a given pathway that contributed to the core enrichment. The dot color displays a gradient reflecting the FDR q-value.

**Supplementary Figure S1.** PRISMA 2020 flow diagram of the study selection process.

**Supplementary Figure S2.** Contingency tables and standardized residuals for the healthNF → cancerNF → CAF trajectory

**Supplementary Figure S3.** Contingency tables and standardized residuals for the healthNF → riskNF → CAF trajectory

**Supplementary Figure S4**. Paired scatterplots for CXCL12 and fibroblast marker genes

**Supplementary Figure S5**. Correlation matrices of gene expression for healthNF → [cancerNF/riskNF] → CAF trajectories

**Supplementary Figure S6.** Identification of differentially expressed genes in the healthNF → cancerNF → CAF trajectory.

**Supplementary Figure S7**. Identification of differentially expressed genes in the healthNF → riskNF → CAF trajectory

**Supplementary Figure S8.** Contingency tables and standardized residuals of CXCR4 expression by clinical-morphological groups of fibroblasts

**Supplementary Figure S9.** Decomposition comparison of different clinical-morphological groups of fibroblasts.

**Supplementary Figure S10.** Contingency table of “phenotype vs CXCR4 level” of fibroblast subtypes
